# Ablation of Wnt signaling in bone marrow stromal cells overcomes microenvironment-mediated drug resistance in acute myeloid leukemia

**DOI:** 10.1038/s41598-024-58860-8

**Published:** 2024-04-10

**Authors:** Hamenth Kumar Palani, Saravanan Ganesan, Nithya Balasundaram, Arvind Venkatraman, Anu Korula, Aby Abraham, Biju George, Vikram Mathews

**Affiliations:** Department of Haematology, Christian Medical College, Ranipet Campus, Vellore, 632 517 India

**Keywords:** Cancer microenvironment, Haematological cancer, Preclinical research

## Abstract

The survival of leukemic cells is significantly influenced by the bone marrow microenvironment, where stromal cells play a crucial role. While there has been substantial progress in understanding the mechanisms and pathways involved in this crosstalk, limited data exist regarding the impact of leukemic cells on bone marrow stromal cells and their potential role in drug resistance. In this study, we identify that leukemic cells prime bone marrow stromal cells towards osteoblast lineage and promote drug resistance. This biased differentiation of stroma is accompanied by dysregulation of the canonical Wnt signaling pathway. Inhibition of Wnt signaling in stroma reversed the drug resistance in leukemic cells, which was further validated in leukemic mice models. This study evaluates the critical role of leukemic cells in establishing a drug-resistant niche by influencing the bone marrow stromal cells. Additionally, it highlights the potential of targeting Wnt signaling in the stroma by repurposing an anthelmintic drug to overcome the microenvironment-mediated drug resistance.

## Introduction

Emerging evidence suggests that bone marrow stromal cells provide a protective niche to leukemic cells against various chemotherapeutic agents^1^. Studies, including ourselves, have emphasized the importance of bone marrow stromal cells in mediating drug resistance in acute myeloid leukemia (AML)^2,3^, and it has been documented that microenvironment-mediated drug resistance (EM-DR) is modulated by anti-apoptotic gene expression signaling and promotion of a quiescent state^4–6^. While several studies have explored the role of the microenvironment on the phenotype of leukemic cells and its impact on drug resistance^4,7–11^, limited data exist on the influence of these leukemic cells on stromal cells and the bone marrow niche.

It is likely that malignant leukemic cells actively modulate the microenvironment to favor leukemic cell survival and proliferation, yet this intricate crosstalk was not studied in detail. A study demonstrated increased activation of AKT, ERK1/2 and STAT3 signaling in the stroma, accompanied by significant upregulation of HES1 and BCL-2 proteins^10^, critical in mediating EM-DR in AML cells. Reports indicate that, in myeloproliferative neoplasm mice models, leukemic cells reduce the number of mesenchymal stem and progenitor cells (MSPCs) while increasing osteoblast numbers, leading to quiescence and disruption of the normal hematopoietic stem cell niche^12,13^. Furthermore, emerging evidence in AML highlights that leukemic cells interacting with bone marrow stromal cells induce stromal cell differentiation towards the osteoblast lineage, primarily driven by BMP-RUNX2-CTGF signaling pathways^14^. Even in an MLL-AF9-driven AML mouse model, MSPCs exhibit skewed differentiation towards osteoblast, accumulating osteoprogenitor cells within the bone marrow^15^. Interestingly, exosomes secreted by AML cells were found to suppress osteoblast differentiation in stromal cells via DKK1 and its inhibition delayed leukemic progression^16^. In contrast, there are reports in AML, where stromal cells displayed an adipogenic differentiation profile through down-regulation of SOX9 and EGR1^17^ and AML cells were shown to inhibit osteoblast mineralization through inhibition of Wnt β-catenin signaling^18^. In addition, studies indicate that leukemic cells alter the vascular niche by increasing its permeability in the bone marrow microenvironment, potentially contributing to disease progression and drug resistance in AML^19^. Collectively, there is growing evidence of marrow alterations within the niche that can induce leukemic cell survival and proliferation. However, the impact of these alterations in the bone marrow microenvironment and stromal cells is not fully understood, mainly their role in drug resistance and the potential for therapeutically targeting such alterations in AML has not been thoroughly evaluated or reported.

Unrelated to leukemia and stromal cell crosstalk, other studies have indicated that the activation of the Wnt pathway (mutated β-catenin) or activation of β-catenin through other transcription factors such as *FOXO1* in osteoblast can result in AML in mice^20,21^. Similarly, it has been demonstrated that mutations in the *DICER1* gene in the osteoprogenitor cells can also induce AML in mice^22^.

This study evaluated the molecular changes stromal cells undergo when interacting with leukemic cells. Our results suggest that leukemic cells prime the stromal cells towards osteogenic potential, which, in turn, can influence resistance to chemotherapeutic drugs through the Wnt signaling pathway. This study emphasizes the potential importance of targeting alterations in the bone marrow microenvironment in the treatment of acute myeloid leukemia, and we demonstrate a possible therapeutic strategy.

## Results

### Leukemic cells induce global changes in the stromal cells upon in vitro co-culture

**Figure 1:**
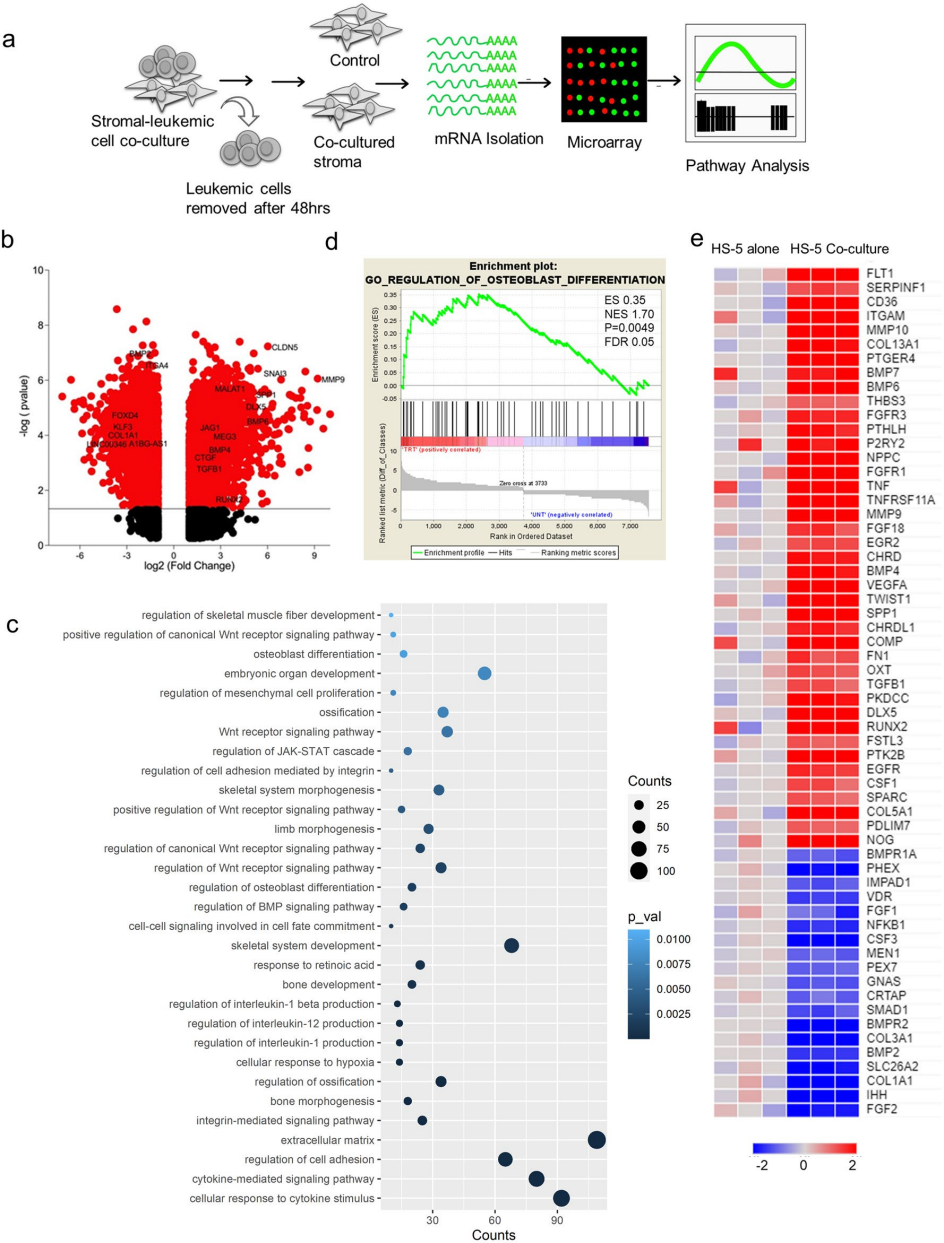
(**a**) Schematic representation of co-culture between stromal cells (HS-5) and leukemic cells (NB4). The stromal cells were subjected to GEP analysis at the end of 48 h. (**b**) Volcano plot showing the differentially regulated genes in stromal cells upon leukemic cell interaction. (**c**) DAVID pathway analysis showed an enrichment of osteoblast differentiation-related genes and Wnt signaling pathways. (**d**) Gene Set Enrichment Analysis (GSEA) analysis indicated an enrichment of osteoblast differentiation pathway in stromal cells upon co-culture. (**e**) Heat map demonstrates osteoblast-associated (differentially regulated) genes enriched in stromal cells upon co-culture with leukemic cells (n = 3).

We have previously reported that stromal cells induce drug resistance in leukemic cells using an in vitro stromal-leukemic cell co-culture system^2,3,23^. To study the effect of leukemic cells on these stromal cells, we used the same system where a normal stromal cell line (HS-5) was co-cultured with leukemic cells (NB4) for 48 h and a global gene expression profile (GEP) of the stromal cell was studied (Fig. 1a). The stromal cells were flow-characterized using CD73 (a marker for stromal cells) for enumeration of residual leukemic cells. The co-cultured stromal cells were shown to be more than 90% purity (CD73 +) before transcriptomic analysis. (Supplementary Fig. 1a, b). The GEP suggested that the transcriptomic profile of stromal cells in co-culture differed from the control (stromal cells alone), indicating that the leukemic cells alter the stromal cells during co-culture. We observed that around 9046 genes were differentially regulated (Fig. 1b). Further pathway analysis of the GEP using the DAVID database suggested an enrichment of bone formation and cellular differentiation of stromal cells towards osteoblastic lineage. Along with these pathways, there was significant enrichment for Wnt signaling, BMP signaling, cell adhesion molecules and cytokines (Fig. 1c). These findings were further confirmed by GSEA analysis, where enrichment of osteoblast differentiation was noted (Fig. 1d) and the differential expression of genes was represented (Fig. 1e). These data were also validated by an independent RNA sequencing experiment in which the stromal cell line was co-cultured with another leukemic cell line (U937) (Supplementary Table S2).

### Stromal cells are primed for osteoblast differentiation by the leukemic cells.

**Figure 2:**
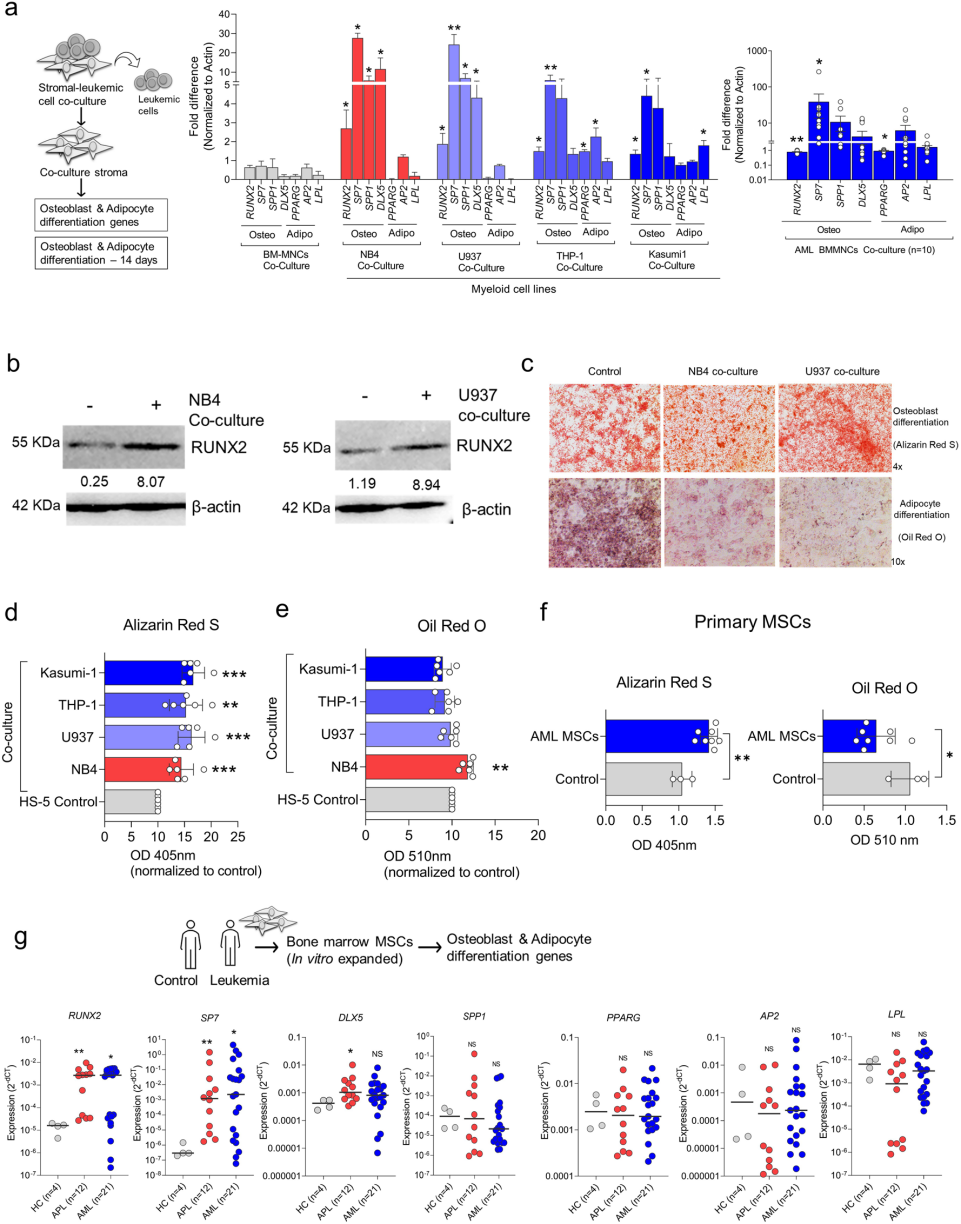
(**a**) QPCR analysis of osteogenic (*RUNX2, SP7, SPP1 & DLX5*) and adipogenic genes (*PPARG, AP2 & LPL*) in stromal cells co-cultured with different myeloid leukemic cell lines (NB4, U937, THP-1 and Kasumi-1) and primary AML cells AML BM-MNCs (n = 10) in comparison to healthy control bone marrow mono-nuclear cells (BM-MNCs) cells co-cultured for 48 h and expression of genes were evaluated by real-time PCR assay (n = 4). Statistical significance was calculated in comparison to control BM-MNCs co-culture. (**b**) Immunoblot demonstrates an increased expression of *RUNX2* (an osteogenic transcription factor) in the stromal cells co-cultured with leukemic cells (NB4 and U937) for 48 h. (**c**) Microscopic picture demonstrating the differentiation of stromal cells into osteoblast and adipocytes after co-culturing with leukemic cells NB4 and U937 cells for 48 h, followed by differentiation of stromal cells for 14 days, and the cells were stained with Alizarin Red S (osteoblast) and Oil Red O (adipocytes). The scale bar is 50 µm. (**d**) Quantifying osteoblast differentiation of stromal cells post co-culture with different myeloid leukemic cells and their colorimetric detection of Alizarin Red S staining using absorbance at 405 nm (n = 5). (**e**) Quantifying adipocyte differentiation of stromal cells post leukemic cell co-culture followed by colorimetric detection of Oil Red O staining using absorbance at 510 nm (n = 5). (**f**) Quantifying the osteoblast and adipocyte differentiation potential of primary AML MSCs (n = 8). (**g**) QPCR analysis of osteogenic and adipogenic genes in primary stromal cells expanded from the bone marrow of healthy control (HC) (n = 4), APL (n = 12), AML (n = 21) (*—*P* = 0.05, **—*P* = 0.001, ***—*P* = 0.0001, ns—not significant).

To validate the GEP analysis, we performed QPCR and western blot assays to verify genes involved in osteoblast differentiation. We noted a significant upregulation of genes involved in osteoblast differentiation (*RUNX2, SP7 & DLX5*) without altering the expression of genes involved in adipocyte differentiation (*PPARG, AP2 & LPL*)) when the stromal cells were co-cultured with myeloid leukemic cell lines and primary cells (Fig. 2a; Supplementary Fig. 1c). The expression of these genes was also evaluated in leukemic cells as a negative control (Supplementary Fig. 1d). In corroboration with QPCR analysis, western blot also demonstrated an increase in RUNX2 (osteogenic transcription factor) in stromal cells during co-culture with leukemic cells (NB4 and U937) (Fig. 2b). For further validation using a functional assay, the stromal cells were co-cultured with different leukemic cells and subjected to differentiation assay. The stromal cells co-cultured with leukemic cell lines demonstrated significantly increased osteoblast differentiation, as indicated by Alizarin Red S staining (Fig. 2c,d). However, there were no changes in differentiation towards adipocytes, as shown by Oil Red O staining (Fig. 2e), with minimal heterogeneity across the cells. In contrast, the primary leukemic cells could not prime the stromal cell line (HS-5) towards osteoblast/ adipocyte differentiation, unlike leukemic cell lines (Supplementary Fig. 2a).

To confirm our findings from cell line models, we used primary stromal cells expanded from the bone marrow of patients with acute leukemia (APL and AML). Subjecting these primary stromal cells to differentiation assays, the AML stroma demonstrated a significant increase in osteoblast differentiation with a simultaneous decrease in adipocyte differentiation potential (Fig. 2f), suggesting that the stromal cells are primed to osteoblast differentiation by the leukemic cells. We also noted that osteoblast differentiation-associated genes (*RUNX2, SP7 & DLX5*) were significantly upregulated across these cells. However, the adipocyte differentiation-associated genes displayed no change in the expression of these genes when compared to healthy controls (Fig. 2g). Additional evaluation of osteocalcin (a marker for bone formation) levels suggests a significantly increased expression in stromal cells post-leukemic co-culture (Supplementary Fig. 2b). Screening of cell adhesion molecules in the co-cultured stromal cells also suggests adhesion molecules related to osteoblast lineage were upregulated (Supplementary Fig. 3).

**Figure 3:**
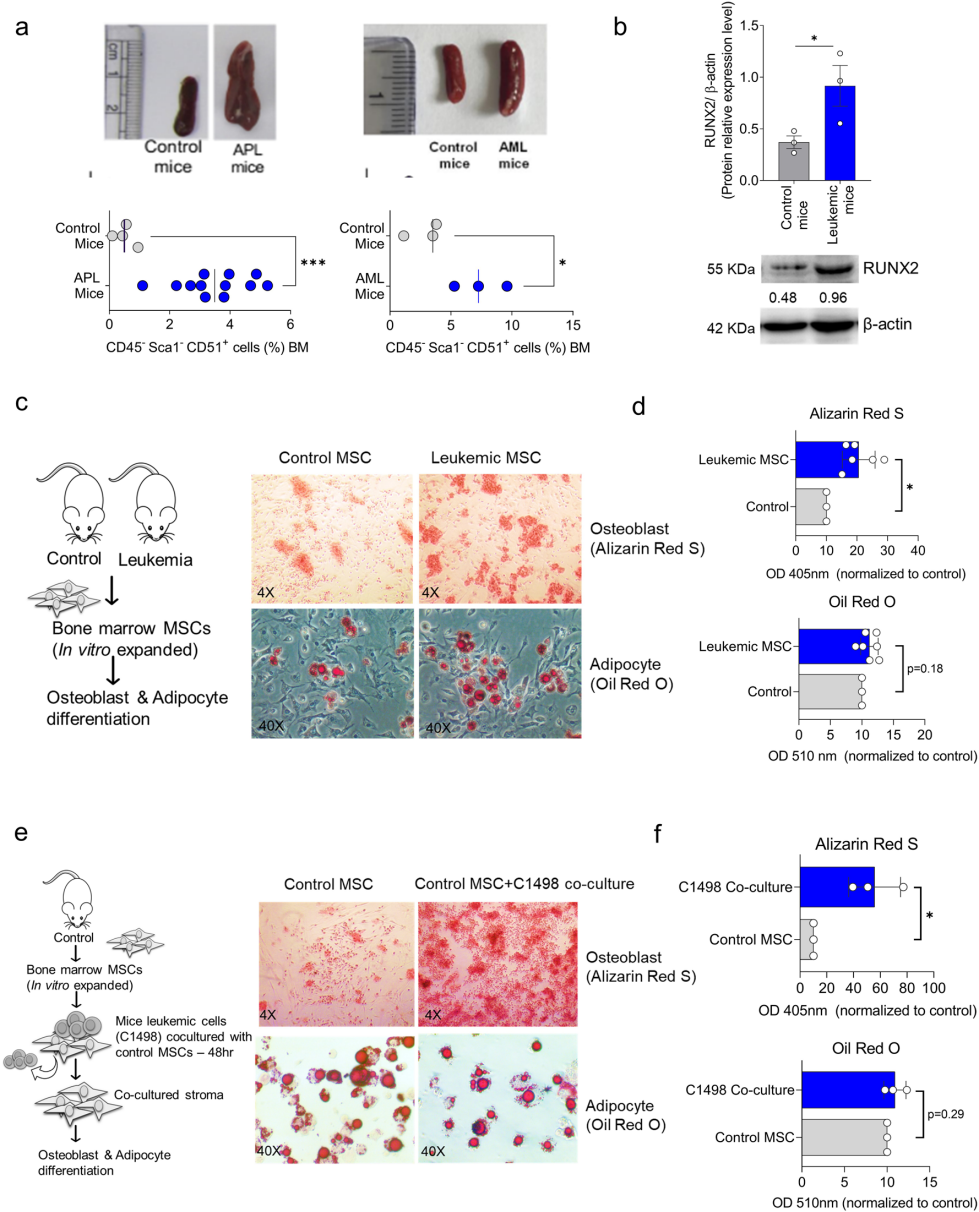
(**a**) APL and AML mice model showing splenomegaly compared to control as well as osteoblast percentage (CD45^−^ Sca1^−^ CD51^+^) cells in bone marrow compared to control mice APL (n = 12) AML (n = 3) on day 28. (**b**) Immunoblot analysis with relative quantification of RUNX2 in bone marrow stromal cells expanded from control and leukemic mice (n = 3). (**c**) Workflow and microscopic picture of Alizarin Red S staining (osteoblast differentiation) and Oil Red O staining (adipocyte differentiation) of the expanded stroma after 14 days of differentiation of leukemic MSCs and control MSCs. The scale bar is 50 µm. (**d**) Quantification of Alizarin Red S and Oil Red O staining demonstrated a substantial increase in osteoblast differentiation (Alizarin Red S staining). In contrast, the adipocyte differentiation (Oil Red O) showed an insignificant variation in control MSCs and leukemic MSCs. e) The MSC expanded from control mice and was co-cultured with mice leukemic cell line C1498 for 48 h, followed by induction for 14 days. The scale bar is 50 µm. The co-cultured MSCs showed an increased osteoblast differentiation without significant change in adipocyte differentiation, as demonstrated in the microscopic picture and colorimetric analysis (f). (*—*P* = 0.05, ns—not significant).

Our in vitro findings were further validated in vivo using two different leukemic mice models [MRP8-PML-RAR transgenic APL mice model (FVB/N strain) and C1498 murine AML cell line model (C57BL/6 strain)]. There was a significant increase in osteoblast percentage (CD45^−^ Sca1^−^ CD51^+)^ in the bone marrow of leukemic mice compared to the control (Fig. 3a). The in vitro expanded mice stroma also demonstrated an increased expression of the osteoblast marker RUNX2, as shown by western blot analysis (Fig. 3b). The differentiation potential of mouse stromal cells exhibited a significant increase in osteoblast differentiation compared to the control, whereas the level of adipocyte differentiation was not substantial (Fig. 3c,d). In addition, we co-cultured the mouse leukemic cell line (C1498) with stromal cells expanded from control mice and evaluated their differentiation potential. Interestingly, the control stromal cells co-cultured with leukemic cells also showed an increased osteoblast differentiation (Fig. 3e,f). The data suggest that leukemic cells prime the stromal cells to commit to differentiation towards the osteoblast lineage.

### Osteoblasts differentiated stroma provides superior protection to leukemic cells compared to undifferentiated or adipocyte differentiated stromal cells

**Figure 4:**
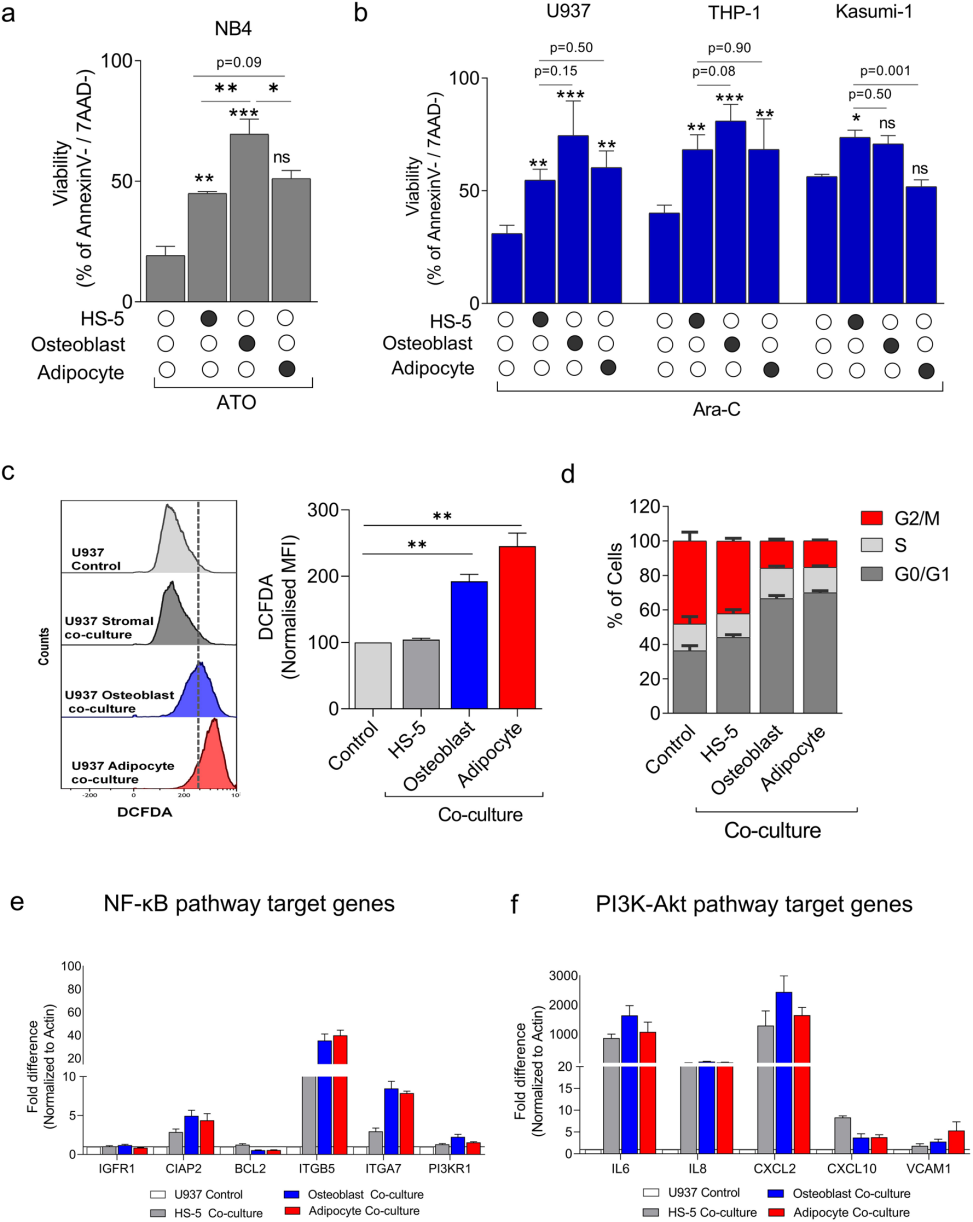
(**a**) Osteoblast differentiated stroma induces a significant protective effect against arsenic trioxide ATO (2 µM) in malignant promyelocytes (NB4) compared to undifferentiated stroma (HS-5) and adipocyte differentiated cells (n = 5). Statistical significance was calculated by comparing the viability of leukemic cells treated with drug alone and leukemic cells co-cultured with different feeder layers, followed by comparison with osteoblast differentiated and undifferentiated cells (**b**) Osteoblasts and adipocytes induce a significant protective effect against cytarabine (Ara-C) (400 ng/ml) in leukemic cells (U937, THP-1 and Kasumi-1) while adipocytes give marginal protection compared to monoculture. Viability was assessed in leukemic cells using an Annexin V/7AAD kit by flow cytometry after 48 h of drug treatment (n = 5). (**c**) Evaluation of ROS levels in leukemic cells post 24 h of co-culture with stromal cells using DCFDA by flow cytometry. (n = 3). (**d**) Cell cycle analysis in leukemic cells post 24 h of co-culture using propidium iodide staining by flow cytometry (n = 6). e) QPCR analysis of NF-κB pathway target genes in leukemic cells post 48 h of co-culture with differentiated and undifferentiated stromal cells compared to control U937 alone (n = 3). f) QPCR analysis of PI3K-Akt pathway target genes (n = 3). (*—*P* = 0.05, **—*P* = 0.001, ***—*P* = 0.0001, ns—not significant).

Since we observed that leukemic cells prime the stromal cells towards osteoblastic lineage, we explored the impact of these primed stromal cells by differentiating them into osteoblast and adipocyte lineages and evaluating their chemo-protective ability. Towards this, stromal cells (HS-5) were differentiated into osteoblast and adipocytes, followed by co-culture with leukemic cells in the presence of chemotherapeutic drugs such as arsenic trioxide (ATO) for promyelocytic cells NB4, daunorubicin (DNR) and cytarabine (Ara-C) for myeloid leukemic cells (U937, THP-1 and Kasumi-1). The viability was assessed at the end of 48 h (Fig. 4a,b). The osteoblast differentiated stroma provides enhanced protection in leukemic cells against ATO and Ara-C compared to adipocyte-differentiated and undifferentiated stromal cells. Though the difference was not statistically significant, the trend toward increase was consistent and reproducible across the leukemic cells evaluated. However, there was a marginal heterogeneity across cells, with a moderate effect in the presence of DNR. (Supplementary Fig. 4). The co-cultured leukemic cells had an increased ROS, and they were in the G0/ G1 quiescence phase of the cell cycle when co-cultured with osteoblast and adipocyte differentiated stroma compared to undifferentiated controls (Fig. 4c,d). These co-cultured leukemic cells possibly depend on cell survival signaling pathways like NF-κB and PI3K-Akt signaling pathway (Fig. 4e,f) demonstrating an increased expression of its target genes, which is consistent with our previous findings in APL and AML^2,3^. The results indicate that leukemic cells, upon stromal co-culture, alter the stromal cells towards the osteoblast lineage to create a drug-resistant niche for survival.

### Wnt signaling is dysregulated in stroma upon co-culture with leukemic cells

**Figure 5:**
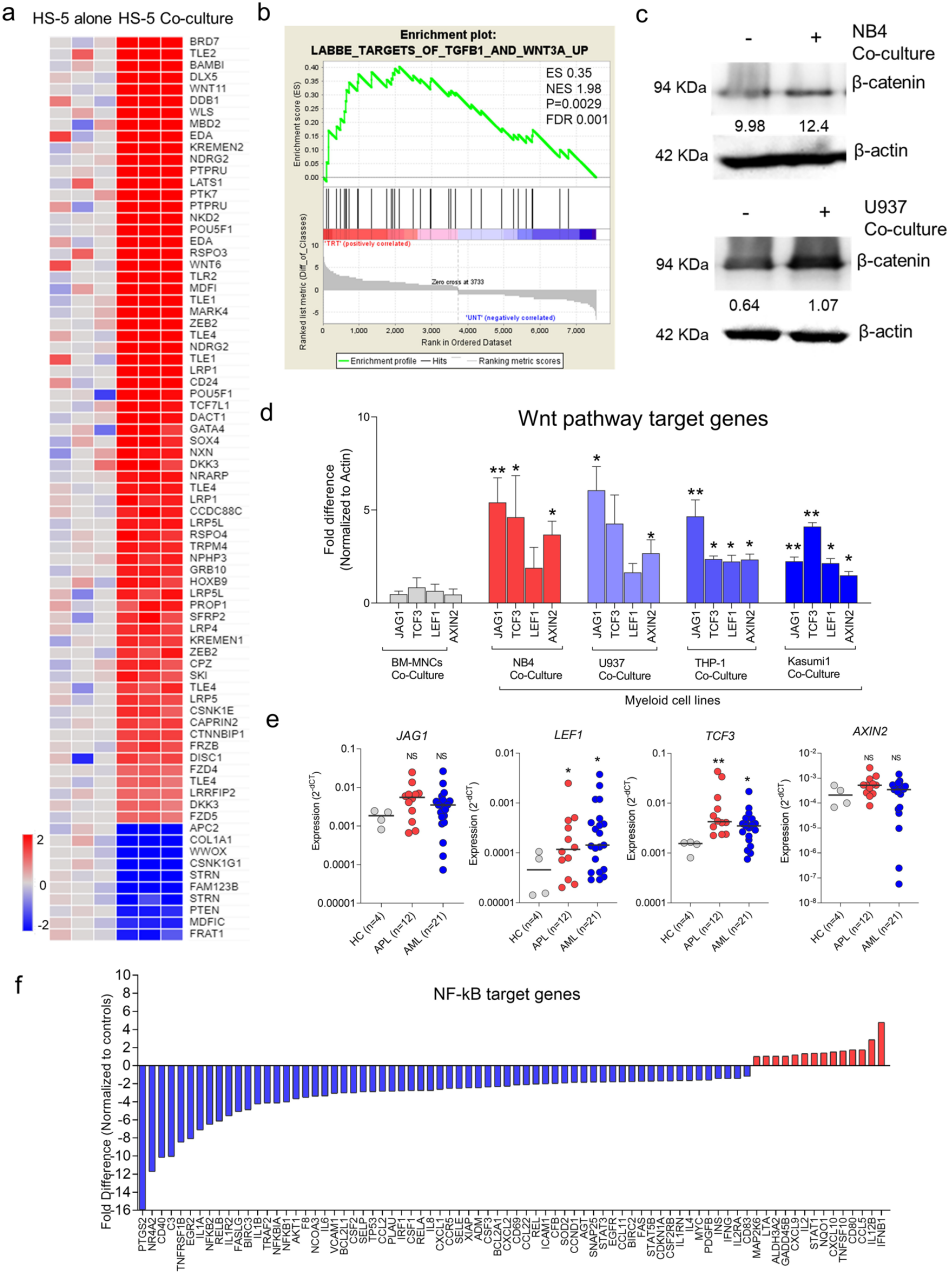
(**a**) Heatmap showing dysregulation of Wnt signaling in stromal cells upon co-culture with leukemic cells from GEP analysis validated by GSEA enrichment analysis (**b**). (**c**) Immunoblot showing stabilization of β-catenin protein in the stromal cells upon co-culture with leukemic cells (NB4 and U937) for 48 h. (**d**) QPCR validation of Wnt signaling genes in stromal cells co-cultured with different leukemic cell lines for 48 h (n = 3). (**e**) QPCR analysis of Wnt signaling genes in primary MSCs from APL (n = 12) and AML (n = 21) in comparison to HC (n = 4). (**f**) Real-time PCR array for NF-κB target genes showing a down-regulation of genes controlled by NF-κB in stomal cells co-cultured with leukemic cells (NB4) (n = 3) for 48 h. (*—*P* = 0.05, **—*P* = 0.001, ns—not significant).

A previous report suggests that the osteoblast priming of stromal cells by leukemic cells mainly involves BMP signaling^14^. Our gene expression profiling also showed that Wnt signaling was enriched in stromal cells upon co-culture with leukemic cells (Fig. 5a,b). It has been established that Wnt signaling is essential for stroma cells to commit to osteoblast lineage in the presence of BMP signaling and, in its absence, stromal cells commit to chondrocyte lineage^24^. Therefore, it was necessary to evaluate the role of canonical Wnt signaling in stromal cells towards drug resistance. The Wnt signaling is activated in co-cultured stromal cells through the stabilization of β-catenin protein, which was confirmed by western blot analysis (Fig. 5c), and its target genes (*JAG1, LEF1, TCF3* & *AXIN2*) was confirmed by QPCR in stromal cells upon co-culture with different myeloid leukemic cells (Fig. 5d). This in vitro data was further validated in primary stromal cells from APL and AML, demonstrating a significant increase in expression of *LEF1* and *TCF3*, with a moderate increase in *JAG1* & *AXIN2* suggestive of variation could be individual and context-dependent (Fig. 5e). Evidence suggests that Wnt activation can antagonize the NF-κB signaling pathway^25,26^. To evaluate this, we performed an NF-κB target gene array and found that most of the NF-κB target genes were downregulated in stromal cells upon co-culture with leukemic cells (NB4) (Fig. 5f). These findings suggest that the Wnt signaling pathway is activated in stromal cells upon co-culture with leukemic cells.

### Inhibition of Wnt signaling in vitro and in vivo reduces the drug resistance phenotype in AML

**Figure 6:**
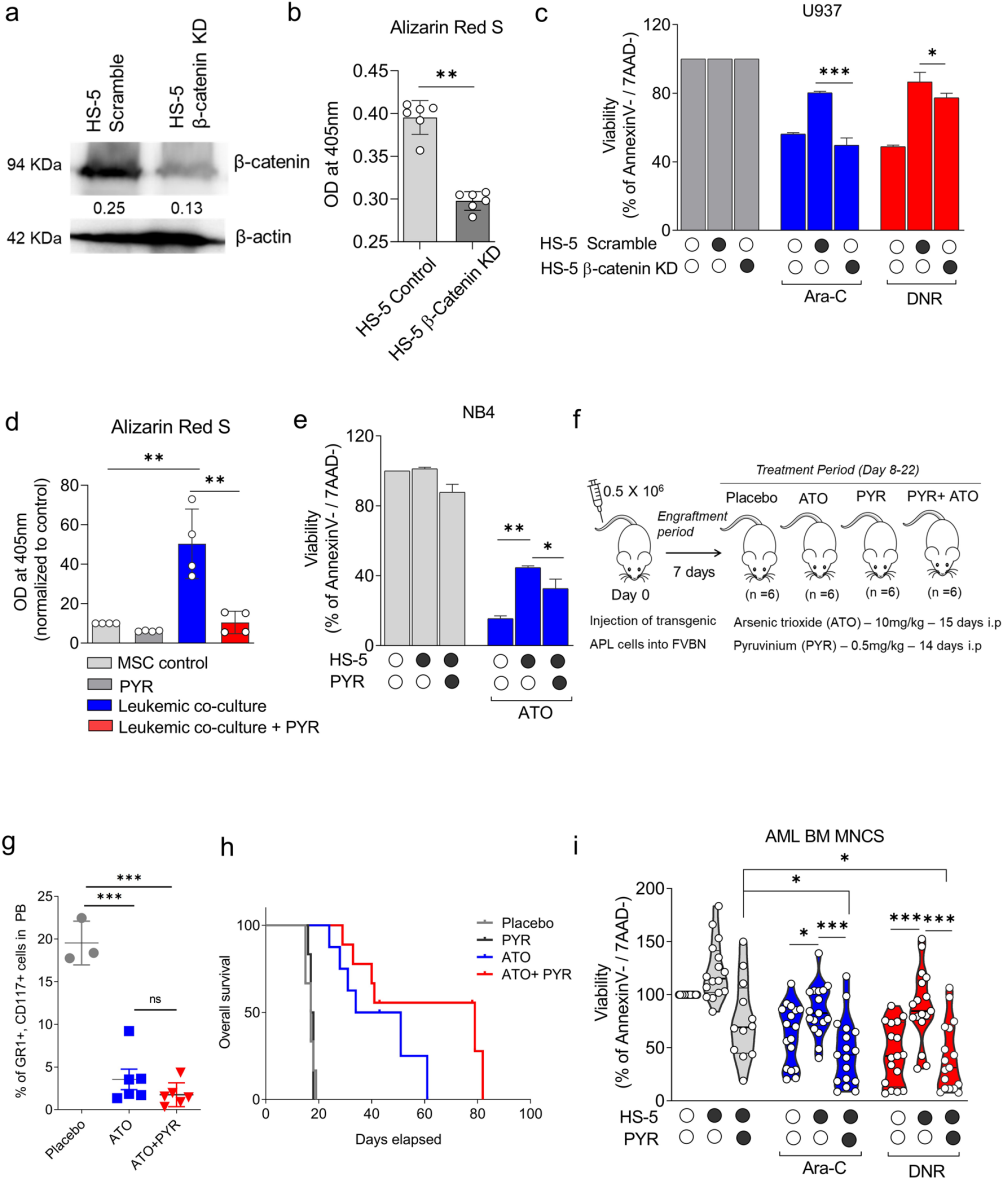
(**a**) Immunoblot analysis demonstrating the knockdown (KD) efficacy of β-catenin in stromal cells compared to control. (**b**) Alizarin Red S staining demonstrates less osteoblast differentiation in HS-5 β-catenin KD cells (n = 6). (**c**) HS-5 β-catenin KD cells reversed the drug resistance in leukemia cells when co-cultured with the knockdown cells compared to scrambled HS-5 cells as shown by annexin V apoptosis assay for 48 h (n = 4). (**d**) Inhibition of the Wnt signaling pathway using a known Wnt inhibitor and an anthelmintic drug, pyrvinium pamoate (PYR), overcomes osteoblast differentiation, as evident by Alizarin Red S staining on MSCs co-cultured with leukemic cells U937 and differentiated to osteoblast for 14 days with PYR at concentration 0.1 µM (n = 4). (**e**) PYR pre-treatment reversed drug resistance in leukemic cells (NB4) upon pre-treatment with PYR, and the viability of leukemic cells was assessed after 48 h (n = 3). (**f**) Workflow of APL mice model on treatment with PYR and ATO. (**g**) PYR, when combined with ATO, reduced the leukemic burden (GR1^+^ CD117^+^ cells) in the APL mice model as evaluated by flow cytometry analysis in peripheral blood on day 28. (**h**) Combination of PYR with ATO in APL mice prolonged the survival of the mice compared to placebo or either of the agents treated alone (n = 6). For survival, a log-rank test was employed to calculate significance. (**i**) PYR pre-treatment effectively overcomes the stromal cell-mediated drug resistance in combination with Ara-C and DNR in AML BM MNCs (n = 17). Each dot represents a patient sample. (*—*P* = 0.05, **—*P* = 0.001, ***—*P* = 0.0001, ns—not significant).

Since Wnt signaling was upregulated in co-cultured stromal cells, we hypothesized it could induce drug resistance in leukemic cells. We knocked down the β-catenin RNA by the shRNA method (Fig. 6a). When the HS-5 cells were knocked down with β-catenin, there was no change in the proliferation of cells (Supplementary Fig. 5), and there was a significant decrease in osteoblast differentiation upon induction, as evidenced by less Alizarin Red S staining (Fig. 6b). These cells have a significantly less cytoprotective effect on leukemic cells from AML chemotherapeutic drugs (Ara-C & DNR) when compared to scramble HS-5 (Fig. 6c). This was further confirmed by Wnt chemical inhibitors. We used an anthelmintic drug, pyrvinium pamoate (PYR), a known Wnt signaling pathway inhibitor^27^. PYR treatment inhibits Wnt signaling in vitro and in vivo (Supplementary Fig. 6) and reduces osteoblast differentiation in stromal cells during leukemic co-culture (Fig. 6d), thereby reverses the drug resistance in leukemic cells (Fig. 6e). PYR pre-treatment in stromal cells abrogated the drug resistance and overcomes the stroma-mediated protective effect in myeloid leukemic cells U937, THP-1 and Kasumi-1 as evidenced by Annexin V apoptosis assay and time-lapse microscopic analysis in U937 demonstrates the increased apoptotic cells in the combination group (PYR + Ara-C) when compared to PYR alone at 48 h (Supplementary Fig. 7). The data was further confirmed by trypan blue staining viability analysis (Supplementary Fig. 8). PYR also showed an additive effect with ATO, Ara-C & DNR in NB4 and U937, respectively, with a synergy score of less than 10 in the presence or absence of stromal cells when the synergy was evaluated using the SynergyFinder tool^28^ (Supplementary Fig. 9). The data validates our hypothesis that the Wnt signaling is upregulated during leukemic cell interaction, which confers drug resistance to leukemic cells against chemotherapeutic agents.

Lastly, our in vitro results were validated in vivo using a transgenic APL mice model, where an increased osteoblast percentage was observed in leukemic mice. Combining PYR with the standard chemotherapeutic drug ATO (Fig. 6f) could significantly reduce the tumor burden in APL mice (Fig. 6g) and increase survival compared to ATO or PYR alone treatment (Fig. 6 h). The efficacy of PYR was also evaluated in mice before or after leukemic cell engraftment. Consistent with our in vitro findings, the APL mice treated with PYR resulted in decreased leukemic burden (Supplementary Fig. 10a, b) as well as osteoblast percentage in bone marrow (Supplementary Fig. 10c). In addition, the PYR treatment also prolonged the survival of mice from a median of 23 days (placebo) to 34 days (when PYR was administered before leukemic engraftment) and by 29 days (when PYR was administered post leukemic engraftment along with ATO) (Supplementary Fig. 10d). However, the combination and the dose utilized for the experiments were not toxic to the normal mice, as evidenced by their survival over three months (Supplementary Fig. 10e). In addition, PYR was also able to overcome the stromal cell-mediated resistance in primary AML cells in combination with cytarabine and daunorubicin (Fig. 6i).

### Impact of lymphoid leukemic cells on stromal cell differentiation and drug resistance

To evaluate the potential of lymphoid leukemic cells in the differentiation of stromal cells, we repeated all the experiments in lymphoid leukemic cell lines SUP B15 (B-ALL), Jurkat E6.1 (T-ALL) and primary ALL cells. Like myeloid leukemic cells, the lymphoid leukemic cells also exhibited a significant upregulation of osteoblast differentiation-associated genes during co-culture (Supplementary Fig. 11a,b), along with increased expression of osteocalcin (Supplementary Fig. 11c). In addition, the functional assay, stromal cell differentiation with ALL cells co-cultured stroma, exhibited a significant differentiation towards osteoblast/ adipocyte lineage (Supplementary Fig. 11d,e). However, the primary ALL cells could not induce differentiation (Supplementary Fig. 11f). Similarly, the primary stroma from ALL patients did not show an increase in osteoblast differentiation. Still, it significantly reduced adipocyte lineage despite a substantial upregulation of osteoblast differentiation genes (*RUNX2, SP7 & DLX5*) (Supplementary Fig. 11g,h).

Consistent with AML data, the ALL-co-cultured stroma also showed a significant upregulation of Wnt target genes (*TCF3*, *LEF1* & *AXIN2*) with elevated expression of *AXIN2* in SUP B15 (Supplementary Fig. 12). However, the differentiated stromal cells had a variable effect on inducing resistance in both ALL cell lines used in this study (Supplementary Fig. 13). Conversely, in the primary ALL cell co-culture, PYR pre-treatment of stromal cells overcomes the resistance exhibited by the stromal cells (Supplementary Fig. 14). Collectively, the data demonstrates that lymphoid leukemic cells, in contrast to myeloid leukemic cells, have a similar but less prominent priming effect on stromal cells to induce the osteoblast differentiation bias and creating drug resistance niche.

## Discussion

The tumor microenvironment is essential in disease progression and drug resistance in cancer cells^29^. Increased inflammation mediated by the cancer cells in this microenvironment plays a central role by inducing various hallmarks of cancers, including angiogenesis, altered metabolism, immune evasion, suppression of cell death, and sustained proliferation^30^. In acute leukemia, numerous studies have described the critical role of bone marrow stromal cells in inducing drug resistance, quiescence, proliferation, and metabolic changes. It nurtures the leukemic cells with various cytokines and cell adhesion molecules to create its physical niche in the marrow^4,7–11^. We have previously reported that the role of stromal cells on drug resistance in myeloid leukemia cells is predominantly mediated by the NF-κB pathway-regulated miRNA axis influencing the autophagy process^2,3^. However, limited studies have highlighted the significant changes in these stromal cells in the leukemic microenvironment. Previous reports suggest that leukemic cells prime the stromal cells towards either osteoblast^14,15^ or adipocyte^17,31^ differentiation. However, the impact of these stromal changes, mediated by the leukemic cells, on drug resistance has not been previously described. A study reported that the stromal cells expanded from AML patients, offering protection like normal stroma. However, the AML stroma is anti-inflammatory in nature and suppresses lymphocyte proliferation^32^. Therefore, this study aims to evaluate the changes in stromal cells interacting with leukemic cells and their impact on drug resistance.

We adopted a GEP approach to identify the global changes in normal stromal cells when co-cultured with leukemic cells. We have established this co-culture system and have previously demonstrated that these stromal cells protect leukemic cells upon drug treatment. Gene expression analysis revealed a comprehensive change in the stromal cells when they interact with leukemic cells, particularly with the differentiation bias of the stroma; we noted that the genes involved in osteoblast differentiation were significantly upregulated in stromal cells. Upon validation, we noted a marginal downregulation of adipocyte genes in stromal cells and no changes of adipocyte genes in primary stroma from acute leukemia patients. This was further confirmed in an independent assay, where we co-cultured stromal cells with different myeloid and lymphoid leukemic cells. These results were similar to the study published by Batula et al.^14^. In addition, when we subjected the stromal cell line or primary stroma to differentiation after co-culturing with leukemic cells, there was a significant increase in osteoblast differentiation. In contrast, the adipocyte differentiation showed heterogeneity across the cells, especially in ALL. However, the primary leukemic cells could not recapitulate this phenomenon of osteoblast differentiation of stromal cell line, and this could be due to a dilution of leukemic cells with contaminant normal mononuclear cells or subtle changes in the phenotype of primary leukemia cells over time in vitro; we have not done adequate experiments to purify the blast to address this further.

We suspect that differentiation of this stroma could be influenced by the loss of the alteration in the stromal cells upon passage, and it could also be influenced by blast numbers, genotype/ phenotype of leukemia, or the stage of the disease. The stromal cells expanded from the marrow of AML mice with an increased leukemia burden and showed a concomitant increase in osteoblast and adipocyte differentiation, with a significant shift towards osteoblast differentiation. This suggests that the leukemic cells prime the stromal cells towards the osteoblast lineage.

To evaluate the correlation between osteoblast differentiation and drug resistance in leukemic cells, the stroma was differentiated into osteoblasts/ adipocytes and co-cultured with leukemic cells, followed by an apoptosis assay as established previously. When differentiated into osteoblast, we noted that the stroma provided increased protection to the leukemic cells compared to adipocytes or control with diverse effects across cells and drugs, especially daunorubicin, have a minimal impact, which warrants further studies. However, the adipocytes also provided protection to leukemic cells. This observation supports the previous study by Ye H et al., which suggested that AML cells can exert drug resistance by extracting fatty acids from the adipocytes in the bone marrow^33^.

Subsequently, we wanted to evaluate the biological pathways involved in this differentiation process. Previous reports^14^ suggest that this stromal cell differentiation can be driven by the BMP signaling pathway via the CTGF molecule. In addition to the upregulation of CTGF and BMP ligands in our GEP data, we found a significant dysregulation of Wnt signaling pathway genes. It has been previously described that the activation of Wnt signaling attenuates PPARγ-mediated adipogenesis, thereby favoring the differentiation of stromal cells into osteoblast^24^. We validated the upregulation of Wnt signaling through β-catenin stabilization, and the genes associated with the Wnt pathway were shown to be upregulated in stromal cells upon co-culture with leukemic cells. To elucidate the potential role of this pathway, we inhibited Wnt signaling using knockdown and chemical inhibitor strategies. We demonstrated that inhibiting Wnt signaling decreased osteogenesis of the stroma upon induction and reversed the drug resistance in AML cells. We validated this in vivo in a transplantable mouse model of APL. Upon treatment with pyrvinium pamoate, an anthelmintic drug known to inhibit Wnt signaling, we observed that treating mice with pyrvinium alone reduced tumor burden and osteoblast percentage and prolonged the survival of APL mice. The combination of pyrvinium with ATO significantly reduced the leukemic burden and modestly increased the survival of APL mice, and this effect was also seen in myeloid and lymphoid leukemic cells when treated with cytarabine and daunorubicin in combination with pyrvinium. The data suggest that inhibiting Wnt signaling can reverse drug resistance in acute leukemia.

Thus, our study demonstrates the role of stromal cell alterations in drug resistance mechanisms in acute leukemia. Our study recognizes that targeting alterations in stromal cells can influence chemotherapy resistance in acute leukemia. This provides a strategy to combat drug resistance by simultaneously targeting stroma and leukemic cells, potentially improving the outcome for patients with acute myeloid leukemia.

## Materials and methods

### Cell lines and primary cells

The human promyelocytic leukemia cell line NB4 (Kind gift from Dr. Harry Iland, Royal Prince Alfred Hospital (Sydney, Australia) with permission from Dr. Michel Lanotte. Myeloid leukemia cell lines (U937, THP-1 & Kasumi-1) and lymphoid leukemic cell lines (Jurkat E6.1 & Sup-B15) and stromal cell line HS-5 were obtained from American Type Culture Collection (ATCC, Virginia, USA). Mycoplasma detection was done once every six months in the laboratory and made sure that the cell lines used were free from mycoplasma-by mycoplasma PCR detection kit (ATCC). Bone marrow samples from acute myeloid leukemia (AML), acute promyelocytic leukemia (APL), and acute lymphoblastic leukemia (ALL) patients were collected during diagnosis before treatment after obtaining written informed consent. Please refer to the Supplementary Excel file S2 for patient clinical information and additional experimental details.

### Ethical approval

This study was conducted in accordance with the principles of the Declaration of Helsinki and approved by the Institutional Review Board (IRB No.8666) (Christian Medical College, Vellore, India). All methods were performed in accordance with the relevant guidelines and regulations.

### Reagents and antibodies

Arsenic trioxide (ATO) was a kind gift from Intas Pharmaceuticals (Ahmedabad, India). Daunorubicin hydrochloride (DNR), Cytosine Arabinoside (Ara-C), and Pyrvinium pamoate (PYR) were obtained from Sigma-Aldrich (Missouri, USA). The antibodies used for immunoblotting β-actin (clone C4), β-catenin (H102), and RUNX2 (M70) were obtained from Santa Cruz Biotechnology (Texas, USA). Secondary antibodies Anti-mouse IgG (#7076) and Anti-rabbit IgG (#7074) linked with horseradish peroxidase (HRP) were from Cell Signaling Technology (Massachusetts, USA). Flow antibodies such as mouse CD45-pacific blue (30-F11), CD117-PE (2B8), Ly6A/E (Sca-1)-FITC (E13-161.7), CD51-PE (RMV-7), Ly6G/6C (GR1)-APC (RB6-8C5), CD11b (MAC-1)-FITC (M1/70) were acquired from BD Biosciences (New Jersey, USA).

### Assays for apoptosis

Leukemic cell lines or primary cells were added (5 × 10^5^ cells/ well) on a layer of stromal cell line (HS-5) or primary stromal cells in 24 well plates. The co-cultured cells were incubated overnight and then exposed to ATO (at the concentration of 2 µM), Ara-C (400 ng/ml), DNR (40 ng/ml) and PYR (0.1 and 1 µM) along with appropriate controls. After 48 h of incubation at 37 °C CO_2_ incubator, the leukemic cells were carefully pipetted out, and their viability was measured using Annexin V/ 7AAD apoptosis assay kit (BD Biosciences) as per manufacturer’s protocol and acquired at Navios flow cytometry (Beckman Coulter, California, USA). CD73 staining/ HS-5 GFP was used to exclude stromal cells if present during acquisition and analysis. The flow data were analyzed using Kaluza Ver 2.1 software (Beckman Coulter). The concentration of the drugs and assay was similar to our previous studies^3^.

### Semi-quantitative real-time PCR

Total RNA was extracted using TRIzol reagent (Thermo Fisher Scientific, Massachusetts, USA). Five hundred nanograms of the extracted RNA were converted into cDNA using a superscript II cDNA kit (Thermo Fisher Scientific). The expression of genes was studied using the SYBR green method using TB Green Premix (Takara Bio, Shiga, Japan). The details of the primer sequence used in this study are stated in Supplementary Table S1. The Ct values were normalized with *GAPDH* or *ACTB,* and the fold differences were calculated using the 2^−∆∆Ct^ method. The NF-κB array (RT^2^ profiler PCR array human NF-κB signaling target. Qiagen, Hilden, Germany, Catalogue No: PAHS-225z) and adhesion molecule array (RT^2^ profiler PCR array human extracellular matrix and adhesion molecule array, Qiagen, Hilden, Germany, Catalogue No: PAHS-013z) was performed according to manufacturer’s instructions.

### Mice model

#### AML mice model

C57BL/6 mice were obtained from Jackson Laboratory (Maine, USA). Both male and female mice at 6 to 8 weeks of age were used in all the experiments. The C1498 AML model was established as described^34^, and the C1498 murine AML cell line was purchased from ATCC. The C1498 cells were cultured in complete DMEM supplemented with 10% fetal bovine serum. Exponentially growing C1498 cells (1 × 10^6^) were suspended in 100 μl PBS and then intravenously injected into the tail vein of recipient mice. These mice developed leukemia and died within 20 days of transplanting the cells.

#### APL mice model

APL cells from the spleen of MRP8-PML-RAR transgenic mice (FVB/N)^35^ were harvested and cryopreserved (a kind gift from Dr. Scott Kogan). APL cells (0.25 × 10^6^ & 0.5 × 10^6^ cells/ mouse) were injected intravenously via the tail vein into genetically compatible FVB/N recipients, as reported previously^36^. Both male and female mice at 6–8 weeks of age were used for the experiments. Leukemic engraftment was evaluated on day 20 in peripheral blood by retro-orbital sampling. Pyrvinium was given as an intra-peritoneal injection at the concentration of 0.5 mg/ kg of mice starting on days -6, -4 and -2 before leukemic engraftment (pre-leukemic protocol) and on days 2, 4 and 6 post-injection of leukemic cells (post-leukemic) and the mice were evaluated for engraftment potential and monitored for survival. For combination therapy in the APL model, the pyrvinium pamoate was given at the dose of 0.5 mg/ kg for 14 days, and arsenic trioxide was given at the dose of 10 mg/ kg for 15 days starting on day 7 post leukemic engraftment period.

The animal study design and all experimental protocols were approved by the Institutional Animal Ethics Committee (IAEC approval number 02/2018 Christian Medical College, Vellore). The study was carried out in compliance with the ARRIVE guidelines. All methods were carried out in accordance with relevant guidelines and regulations.

### Statistical analysis

Statistical analyses were conducted using GraphPad Prism 7.0 software (GraphPad Software, CA, USA). All data points were represented as mean ± SD. A two-tailed Student’s t-test was used to compare mean values between the two groups. One-way ANOVA was used for experiments where multiple groups were compared with the control group. *P* values < 0.05 were considered statistically significant. The *P-*values were denoted as *—*P* < 0.05, **—*P* = 0.001, ***—*P* = 0.0001, ns—not significant.

For further details and methods, please refer to supplementary information.

### Supplementary Information

Supplementary Information 1.

Supplementary Information 2.

## Data Availability

The datasets generated and analyzed during the current study are available in the NCBI GEO database (GEO accession number GSE124987). Other data supporting the findings in this study are available within the manuscript, as well as supplementary information. Any further details will be available upon request.
