# Supplementary material for: Ablation of Wnt signaling in bone marrow stromal cells overcomes microenvironment-mediated drug resistance in acute myeloid leukemia
Source: Sci Rep. 2024 Apr 10;14:8404. doi: 10.1038/s41598-024-58860-8 (PMC11006665; doi:10.1038/s41598-024-58860-8)
Supplement: Supplementary file 1 — Supplementary Information 1. [file 41598_2024_58860_MOESM1_ESM.docx]

**Ablation of Wnt signaling in bone marrow stromal cells overcomes microenvironment-mediated drug resistance in acute myeloid leukemia.**

Hamenth Kumar Palani*, Saravanan Ganesan*, Nithya Balasundaram, Arvind Venkatraman, Anu Korula, Aby Abraham, Biju George, Vikram Mathews.

* Contributed equally as co-first authors

Department of Haematology, Christian Medical College, Vellore, India.

**Supplementary Information**

**Supplementary methods**

## Mesenchymal stromal cells expansion and differentiation

Mesenchymal stromal cells (MSC) were expanded from a fraction of bone marrow samples obtained from patients who underwent a bone marrow test as part of a diagnostic procedure. The control MSCs were obtained from Non-Hodgkin’s Lymphoma (NHL) patients who underwent a staging marrow where no bone marrow abnormality was detected. The samples were obtained after getting written informed consent. MSCs were expanded from bone marrow mononuclear cells using alpha-MEM media (Thermo Fisher Scientific, Massachusetts, USA) with 10% fetal bovine serum, 100 units/ ml penicillin, and 100 ug/ ml streptomycin (pen-strep), L-Glutamine, β-mercaptoethanol (Thermo Fisher Scientific) in 37° C incubator with 5% CO_2_.

For osteoblast and adipocyte differentiation, the MSCs from passage 2 were used for differentiation. The media for osteoblast differentiation (10% alpha-MEM with pen-strep, L-glutamine, 10 nM dexamethasone, 10 mM β-Glycerophosphate and 50 µg/ml of ascorbic acid) was added for MSC and cultured for 14 days with replacement of differentiation media every three days. Similarly, for adipocyte differentiation, adipogenic differentiation media (10 % alpha-MEM with pen-strep, L-glutamine, 1 µM dexamethasone, 10 µM 3- Isobutyl-1- methylxanthine, 0.1mM Indomethacin, 10 µg/ml Insulin) was used for 14 days with replacement of differentiation media every three days. The osteoblast and adipocyte differentiation were confirmed using Alizarin Red S and Oil Red O staining, respectively. Alizarin Red S staining was quantified using 0.5 N HCL with 5% SDS, and the absorbance was measured at 405nm; the Oil Red O stain was dissolved using isopropanol, and the absorbance was measured at 510nm.

**Murine cell collection**

To determine the percentage of osteoblast, the mice's femur and tibia were collected and cleaned of soft tissues, the bone marrow was flushed with a 25-gauge needle, and the resulting bones were crushed using a mortar and pestle. The cell suspension was filtered using a 40 μm pore size cell strainer. The RBCs were lysed using ammonium chloride buffer (ammonium chloride 8.2g & Sodium bicarbonate 0.84g in 1-litre distilled water with pH 7.4) for 10 minutes, followed by PBS wash. The cells were stained with flow cytometric antibodies, followed by wash and acquisition in flow cytometry. The flow markers CD45^-^ Sca1^-^ CD51^+^ cells were used to determine osteoblast percentage determination in bone marrow. CD117^+^ GR1^+^ cells determine the leukemic burden/ engraftment in the APL model.

To isolate the mice stromal cells, the control and leukemic mice were euthanized, the femur and tibia were flushed and seeded in 10% DMEM supplemented with pen-strep, L-Glutamine, β-mercaptoethanol and incubated in a hypoxic chamber. The media was changed every three days once the stroma had expanded. Stromal cells from passages 2 or 3 were used for the study.

**Gene expression profiling**

Briefly, the stromal cells (HS-5) were cultured in DMEM media. The leukemic cells (NB4) were co-cultured with and without stromal cells for 48 hours before gene expression analysis. Three independent experiments were carried out to serve as biological triplicates for microarray analysis. The HS-5 cells (control) and the HS-5 co-cultured with leukemic cells (treated) were compared to generate statistically significant differentially expressed genes. The labeled RNAs were hybridized to Agilent Human Whole Genome 8 x 60K Gene Expression Array (AMADID: 039494), and the Image analysis was done using Agilent Feature Extraction Software Version 10.5.1.1 to obtain the raw data. Normalization and statistical analysis of the microarray data were done using GeneSpring GX (Agilent Technologies, California, USA) using the 75^th^ percentile shift. The fold difference was calculated by comparing treated samples with control samples. The student t-test and p-value were computed using the volcano plot algorithm. Differentially regulated genes were clustered using hierarchical clustering to identify significant gene expression patterns. Genes were classified based on functions and pathways using the biological interpretation tool Biointerpreter (Genotypic Technology, Bengaluru, India). The differentially regulated gene lists were subjected to gene set enrichment analysis using GSEA v. 2.0.14 software to detect statistically significant genes enriched in the hallmark module (1).

**Flow cytometric analysis of osteocalcin.**

The intracellular levels of osteocalcin were assessed using PE-conjugated mouse anti-human osteocalcin antibody, clone R14-707 (BD Biosciences, New Jersey, USA) by flow cytometry. Briefly, the leukemic cells at the concentration of 5 x 10^5^ cells/ well were co-cultured with a layer of stromal cells (HS5-GFP) for 48 hours, and the leukemic cells were fixed and permeabilized using a fixation/permeabilization kit (BD Biosciences) followed by staining with osteocalcin antibody according to the manufacturer protocol.

**ROS assay**

The levels of reactive oxygen species (ROS) were determined using 2ʹ,7ʹ-Dichlorofluorescin Diacetate (DCFDA) as per the manufacturer’s instructions (Sigma-Aldrich, Missouri, USA). The leukemic cells (U937) at the concentration of 5 x 10^5^ cells/well were co-cultured with/ without stromal cells in 24 well plates for 24 hours, and then the leukemic cells alone were taken for ROS analysis by staining the cells with 1 µM concentration of DCFDA for 10 minutes using plain RPMI media and acquired by flow cytometry.

**Cell cycle analysis**

The leukemic cells (U937) at the concentration of 5 x 10^5^ cells with or without co-culture with stromal cells were washed with sterile PBS and incubated with cell cycle buffer (trisodium citrate – 0.025 g, Nonidet P40 – 75 μl, RNAse – 20 μg/ml and Propidium iodide – 20 μg/ml) for 10 minutes in the dark and then acquired by flow cytometry. The data was analyzed using Kaluza Ver 2.1 software (Beckman Coulter, California, USA).

**Immunoblots**

HS-5 homogenates were obtained by cell lysis in RIPA buffer (Sigma-Aldrich) with complete protease inhibitors (Roche, Basel, Switzerland). Nuclear extracts were taken from cells using a NE-PER kit (Thermo Pierce, Illinois, USA) according to the manufacturer’s protocol. The lysates and elutes were analyzed in SDS-PAGE. After protein transfer to a nitrocellulose membrane, membranes were blocked with non-fat dry milk (5 %, 2 hours), followed by incubation with primary and secondary antibodies. The standard chemiluminescence method detected the protein bands using SuperSignal West Femto (Thermo Scientific).

**Lentivirus-mediated knockdown.**

The shRNA for scramble and β-catenin was purchased from Sigma (TRC cloning vectors - Sigma). The plasmids were amplified, and lentiviral particles were generated according to standard protocols. The transduction efficacy was measured through GFP-positive cells, which acted as a positive control. The lentiviral particles generated were transduced on HS-5 cells for 6 hrs, and the media was changed, allowing the cells to grow in complete media for 24 hrs, followed by treatment of the stromal cells with puromycin (1mg/ ml). The transduced cells were selected for two weeks in puromycin media, and western blot assays confirmed the knockdown.

**Time-lapse microscopy**

The leukemic cells (U937) 2 x 10^4^ / well were co-cultured on the layer of stromal cells (HS-5 GFP) pretreated with PYR, followed by treatment with or without Ara-C and the interactions were immediately captured using time-lapse microscopy (Bio station IM-Q, Nikon, Tokyo, Japan). The entire experiment was done using a specialized Hi-Q4 culture dish, and the interactions were captured over 48 hours; the images were processed by Bio station IM-Q software (Nikon).

**Trypan blue assay**

The trypan blue exclusion assay was used to distinguish live (unstained) and dead cells (trypan blue stained), and the number of viable/ dead cells was estimated using the DeNovix cell counter according to the manufacturer’s protocol (DeNovix, Delaware, USA).

**Cell proliferation assay**

The stromal cells (HS-5 & HS-5 β-catenin KD) 1 x10^6^ cells/ well were stained with Cell Trace Violet dye (Thermo Fisher Scientific) as per the manufacturer's protocol. The dye intensity was evaluated at 48 hours and 72 hours post-staining by flow cytometry.

**Synergy evaluation**

The leukemic cells NB4 and U937 were co-cultured with stromal cells HS-5 and further treated with increasing concentrations of drugs ATO (0.5, 1 and 2 µM), Ara-C (100, 200 and 400 ng/ ml), DNR (10, 20 and 40 ng/ ml), PYR (0.1, 1 and 2 µM) for 48 hours. The viability was assessed using Calcein-AM staining (BioLegend, California, USA) as per the manufacturer's protocol. The synergy of PYR with different chemotherapeutic drugs (ATO, Ara-C & DNR) was evaluated using the SynergyFinder tool (2).

**Supplementary Figure 1: Flow cytometric characterization of stromal cells post co-culture - gene expression profiling & QPCR analysis.**

**
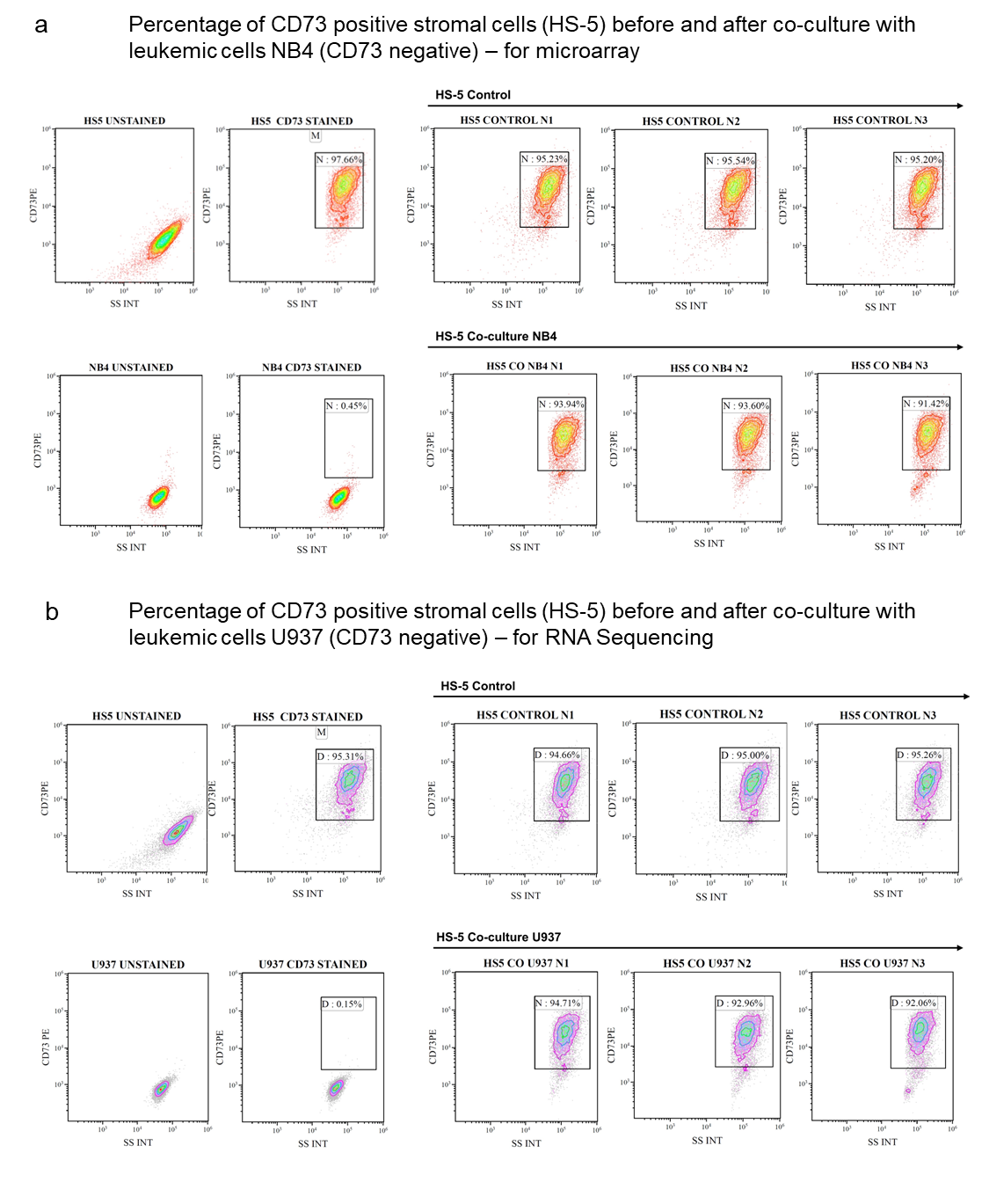
**


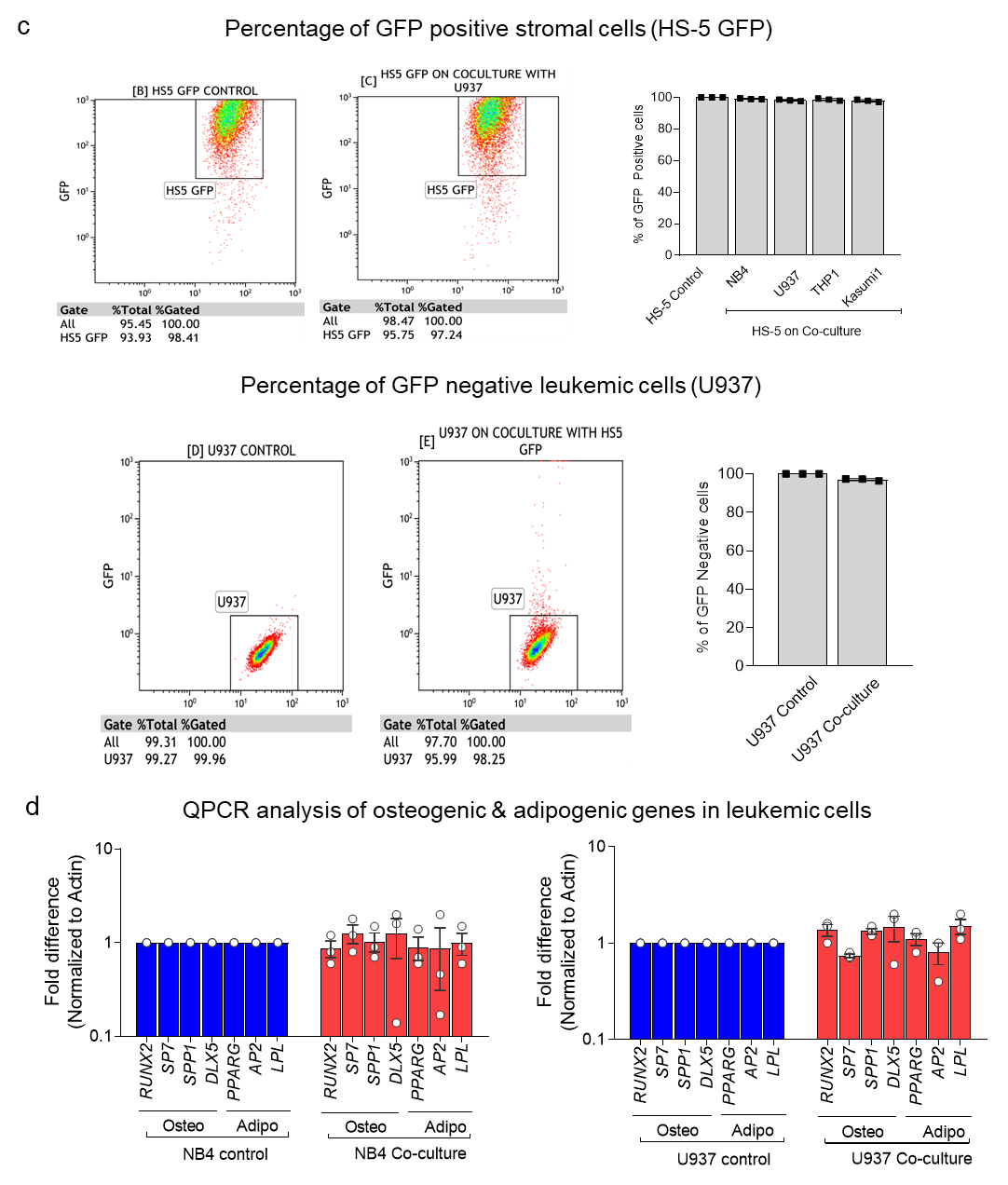


a) and (b) Flow cytometric scatter plots of stromal cells (HS-5) stained with CD73 PE post-co-culture with NB4 and U937 before microarray and RNA Sequencing analysis (n=3). c) Representative scatter plots demonstrate the percentage of GFP positive cells (HS-5 GFP) upon co-culture with leukemic cells (U937) in comparison to control followed by a quantitative bar graph showing the percentage of GFP positive cells (HS-5 GFP) when co-cultured with different leukemic cells demonstrating the purity of stromal cells post co-culture before QPCR analysis. The purity of leukemic cells was assessed by evaluating the percentage of GFP-negative leukemic cells U937. d) QPCR analysis of osteogenic and adipogenic genes in leukemic cells (NB4 & U937) with or without HS-5 co-culture (n=3) as negative control for *figure 2a*.

**Supplementary figure 2. Differentiation potential of stromal cells upon co-culture with primary leukemic cells & osteocalcin expression in co-cultured stromal cells.**

**
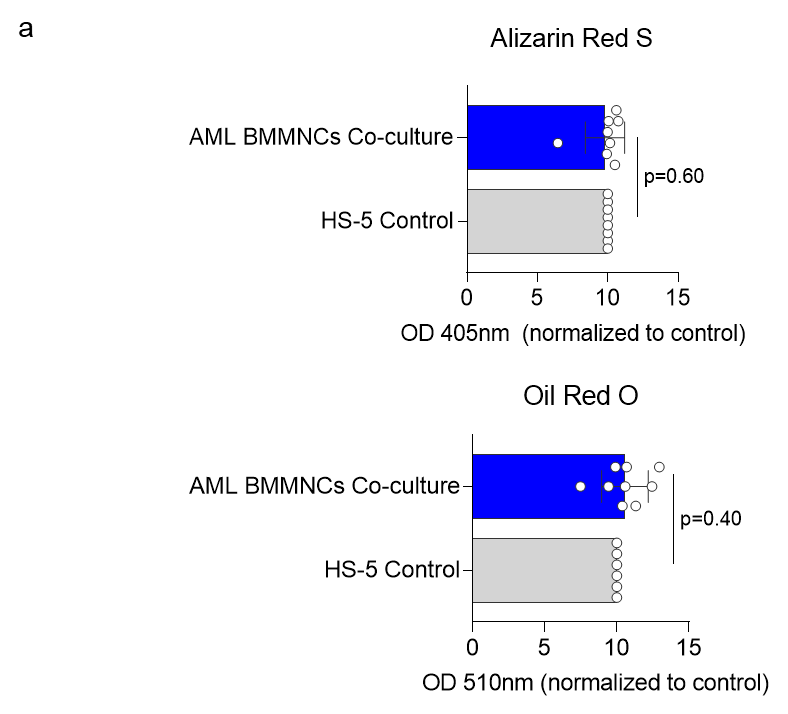
**


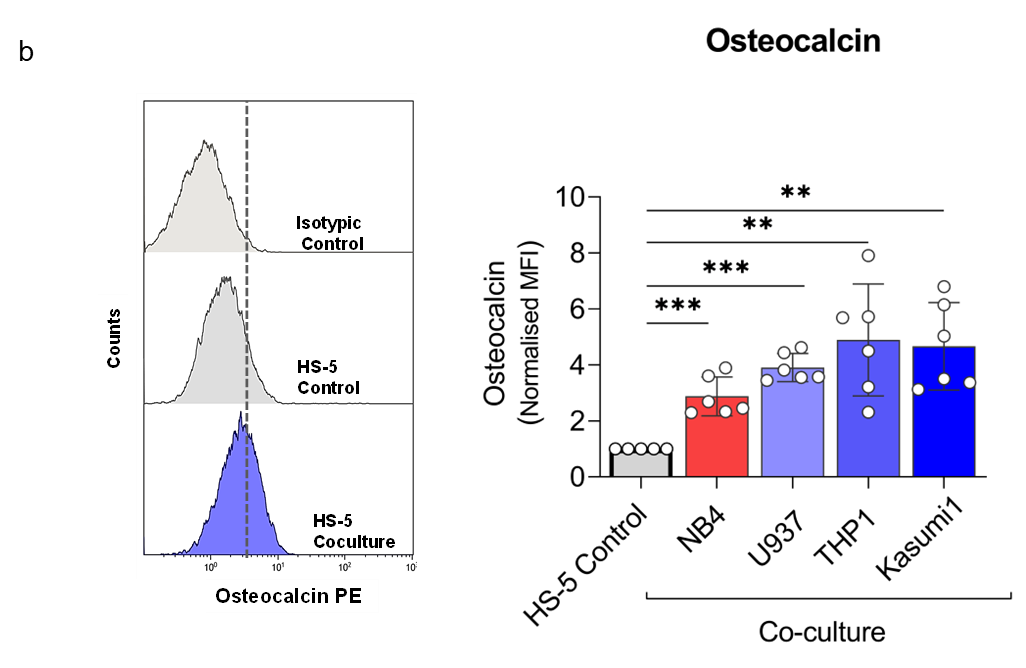


a) Colorimetric analysis of Alizarin Red S (osteoblast differentiation) and Oil Red O staining (adipocyte differentiation) of stromal cells post 48 hrs of co-culture with primary AML cells (n=8) followed by induction for 14 days. b) Illustrative histogram plot showing the increased osteocalcin levels in stromal cells post 48 hrs of co-culture with leukemic cell (U937). Quantitative bar graph demonstrating the mean fluorescence intensity (MFI) of osteocalcin in stromal cells post 48 hrs of co-culture with different leukemic cells. (*-P=0.05, ** - P = 0.001, ***- P=0.0001, ns- Not significant).

**Supplementary figure 3. Differentially expressed adhesion molecules in stromal cells post-co-culture with leukemic cells.**


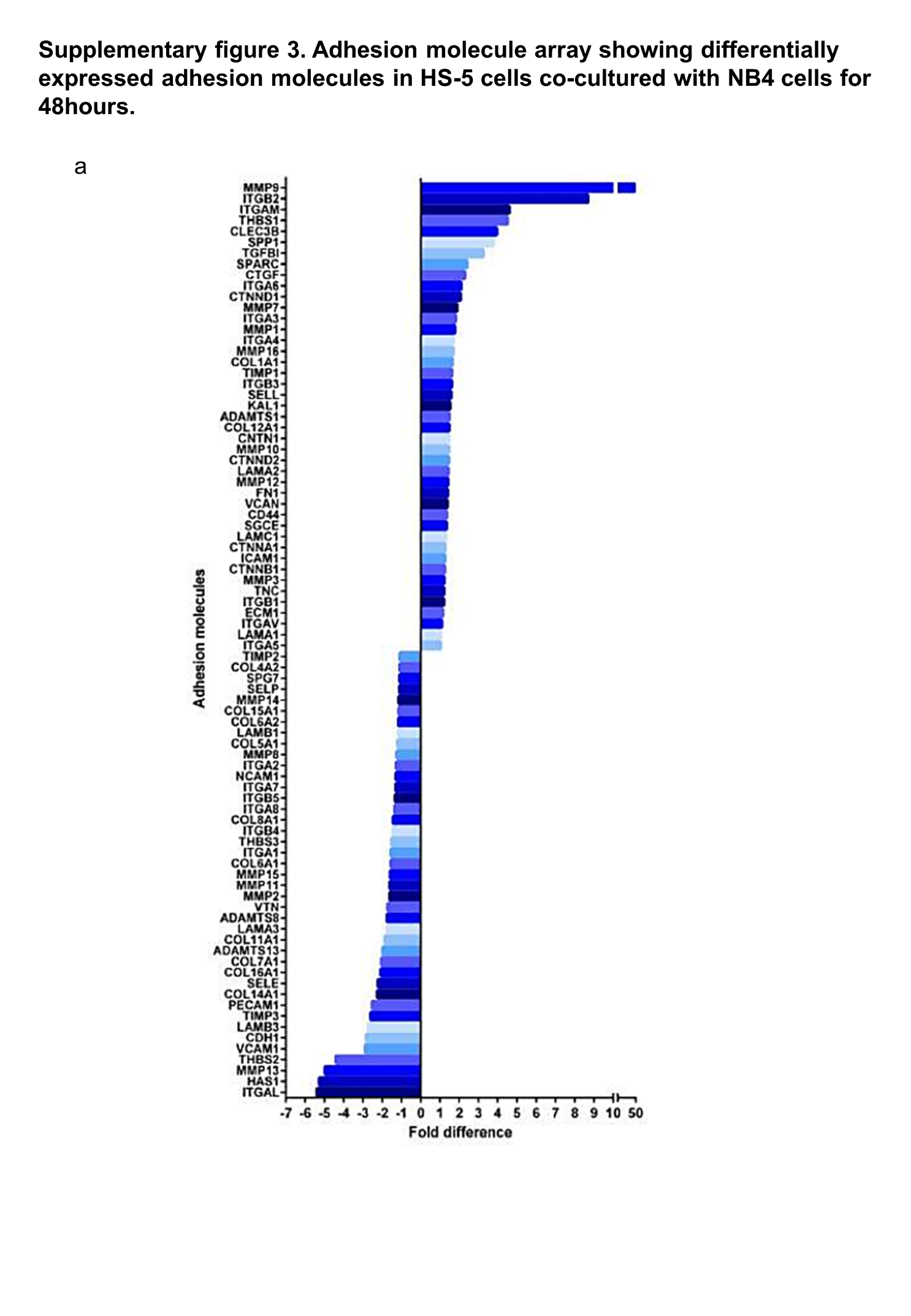


a) QPCR analysis demonstrating the array of adhesion molecules in stromal cells (HS-5) upon co-culture with leukemic cells (NB4) for 48 hrs.

**Supplementary figure 4. Viability of myeloid leukemic cells against daunorubicin in the presence of differentiated stromal cells.**

Viability of myeloid leukemic cells U937, THP-1 and Kasumi-1 upon co-culture with osteoblast and adipocyte differentiated stromal cells followed by treatment with daunorubicin (40ng/ml) compared to control. The viability was assessed after 48 hrs using annexin V 7AAD staining by flow cytometry (n=5).

**Supplementary figure 5. Proliferation ability of HS-5 and HS-5 β-catenin knockdown cells.**

**
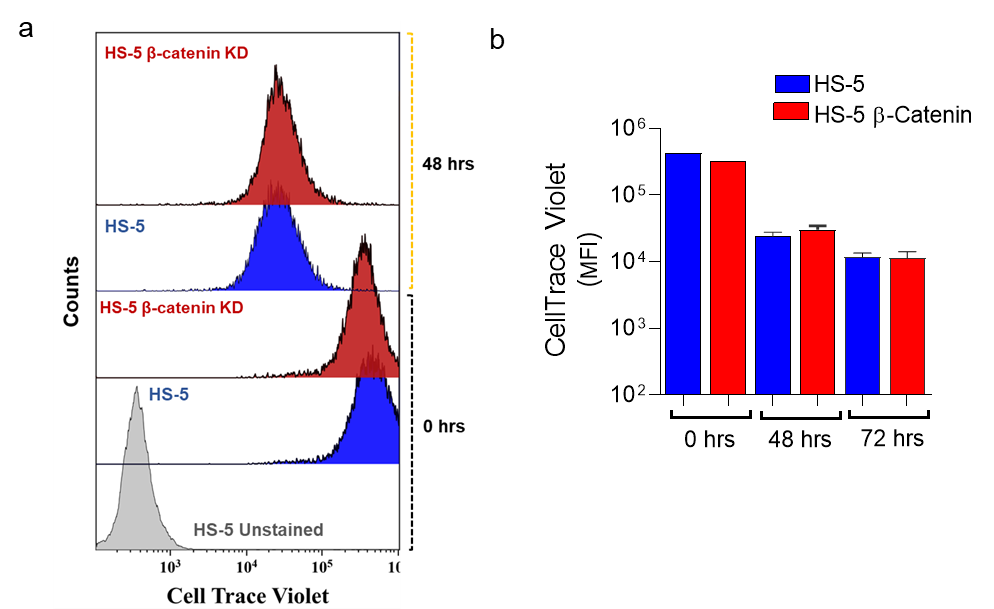
**

a) Illustrative histogram plot demonstrating the intensity of Cell Trace violet staining of HS-5 and HS-5 β-Catenin knockdown cells indicating no difference in proliferation of cells evaluated at different time points 48 hrs. b) Bar graph showing the quantified mean fluorescence intensity MFI (n=3).

**Supplementary figure 6. Pyrvinium treatment inhibits Wnt signaling *in vitro* and *in vivo*.**

**
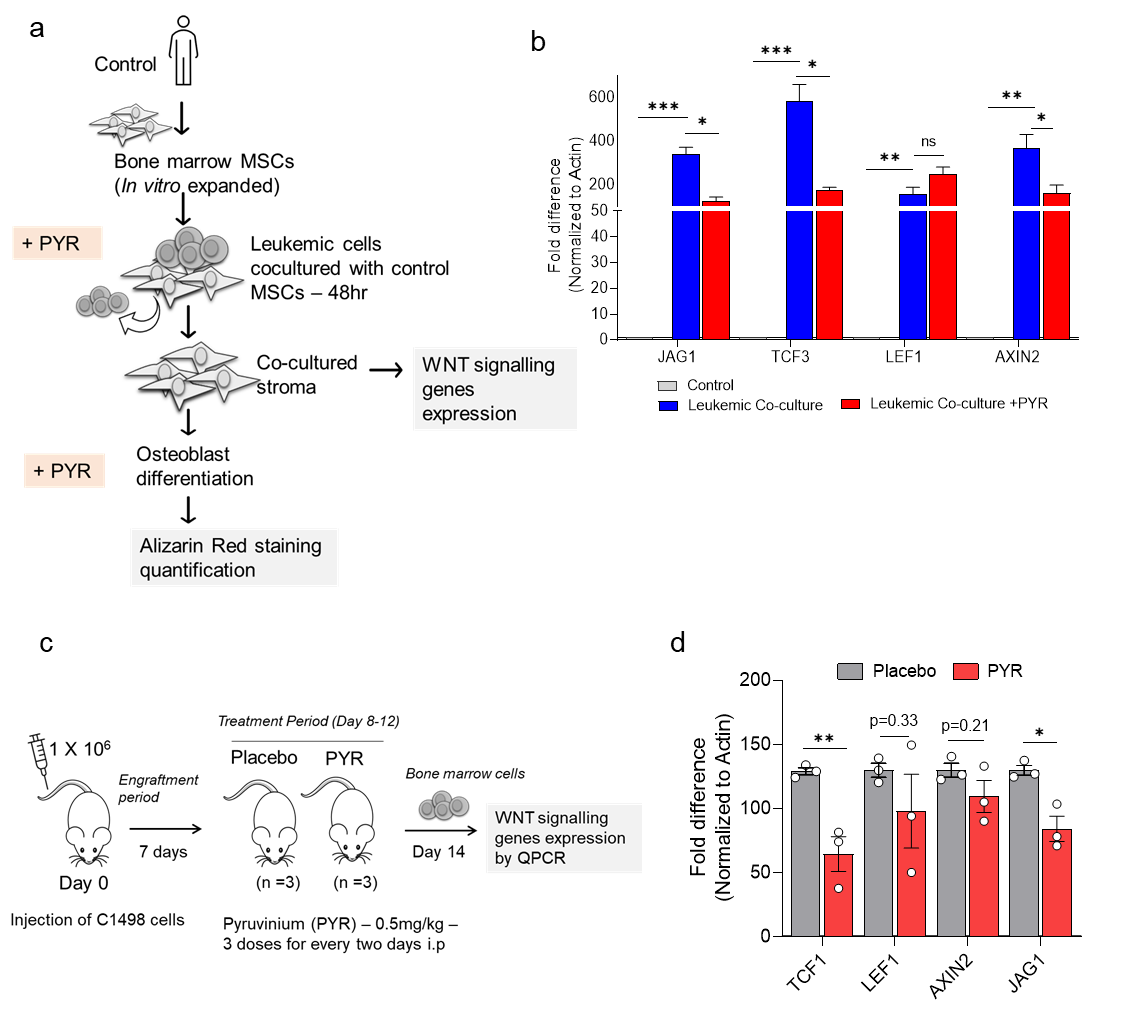
**

a) Schematic *in vitro* experimental plan for treatment of PYR on leukemic and stromal cell co-culture. b) QPCR analysis of Wnt target genes on co-cultured stroma upon PYR treatment (n=4). Osteoblast differentiation potential (Alizarin Red staining) data was demonstrated in *figure 6d. c)* Illustrative *in vivo* experimental plan for C1498 AML mice model, where the mice were treated with three doses of PYR and evaluated its Wnt expression in bone marrow cells. d) QPCR analysis of Wnt target genes in placebo and PYR-treated mice (n=3).


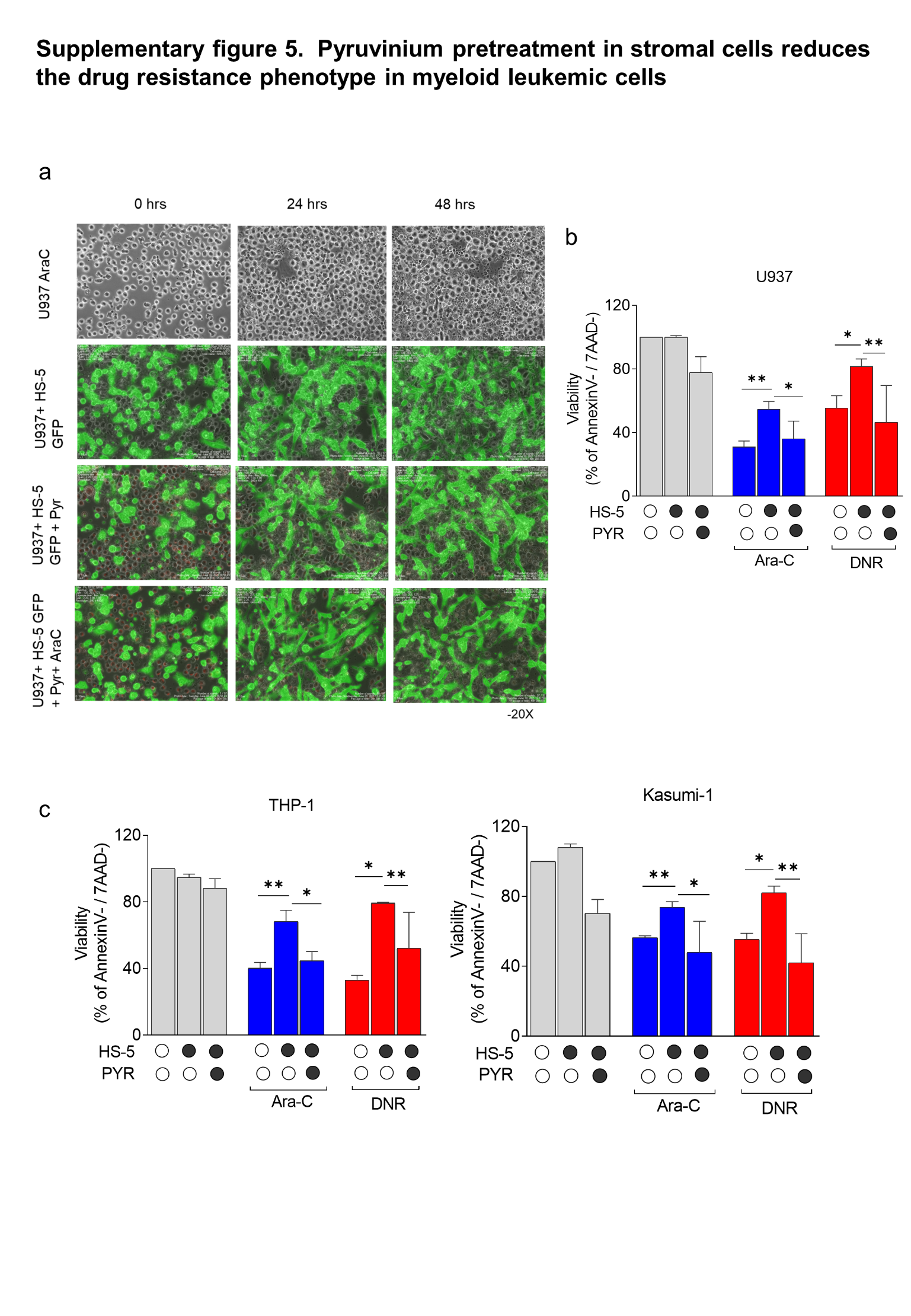
**Supplementary figure 7. Pyrvinium pre-treatment in stromal cells reduces the drug resistance phenotype in myeloid leukemic cells.**

a) Snapshots of live cell imaging analysis of leukemic cells (U937) co-cultured with stromal cells (HS5-GFP) pretreated with/ without PYR (12 hrs) followed by treatment with Ara-C for 48 hrs. (b) and (c) viability of myeloid leukemia cells (U937, THP-1 and Kasumi-1) upon co-culture with stromal cells treated with PYR followed by Ara-C and DNR suggesting that the PYR pre-treatment effectively overcomes the stroma-mediated drug resistance (n=4).

**Supplementary Figure 8: Pyrvinium pretreatment in stromal cells reduces the drug resistance phenotype in myeloid leukemic cells – Trypan blue staining.**


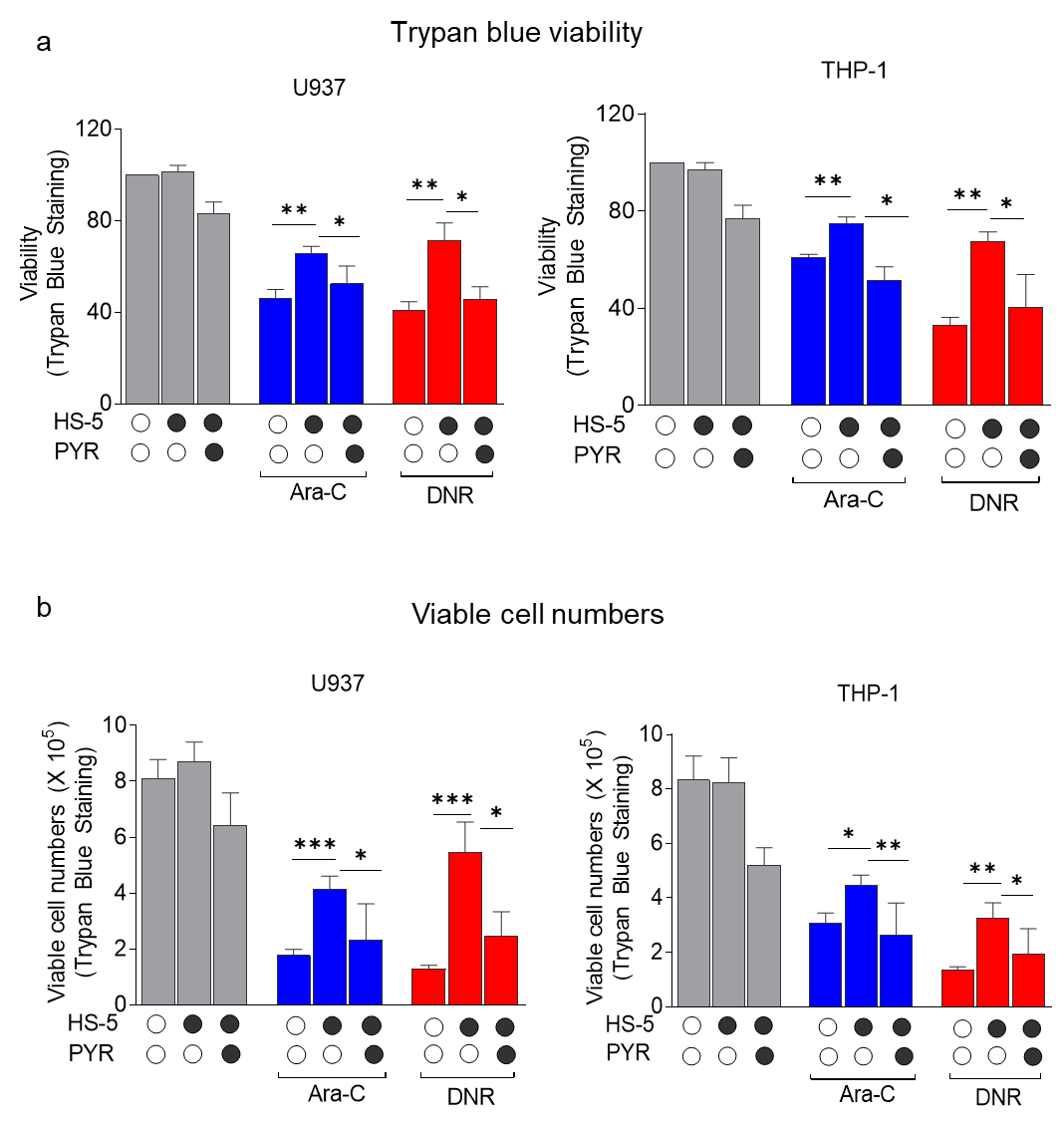


a) and b) viability of myeloid leukemia cells (U937 and THP-1) upon co-culture with stromal cells treated with PYR followed by Ara-C and DNR validating that the PYR treatment effectively overcomes the chemo-protective effect (n=4). The trypan blue exclusion assay was used to distinguish live (unstained) and dead cells (trypan blue stained), and the number of viable/ dead cells was estimated using the DeNovix cell counter.

**Supplementary figure 9: To evaluate the synergy of pyrvinium with AML chemotherapeutic drugs in co-cultured leukemic cells.**

**
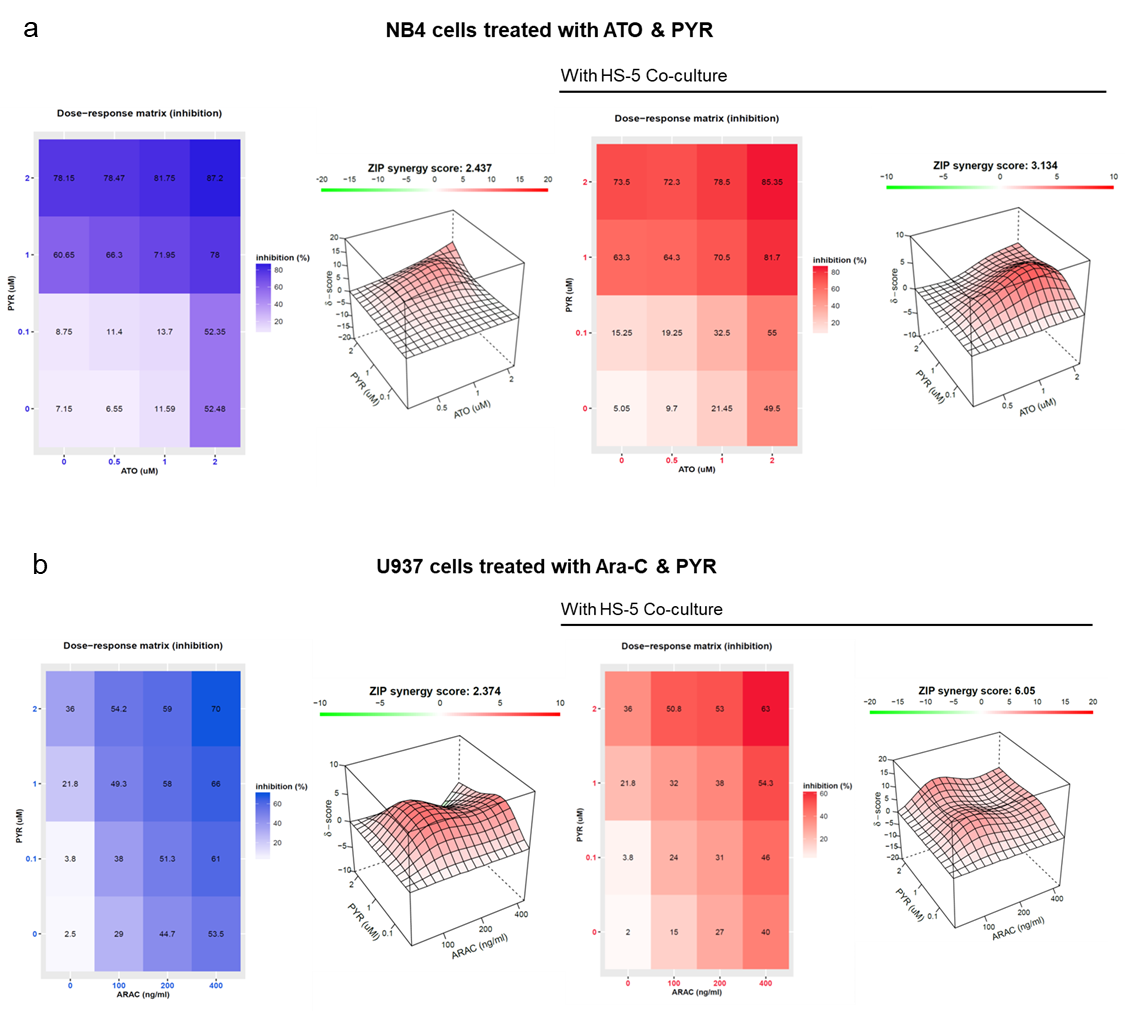
**

**
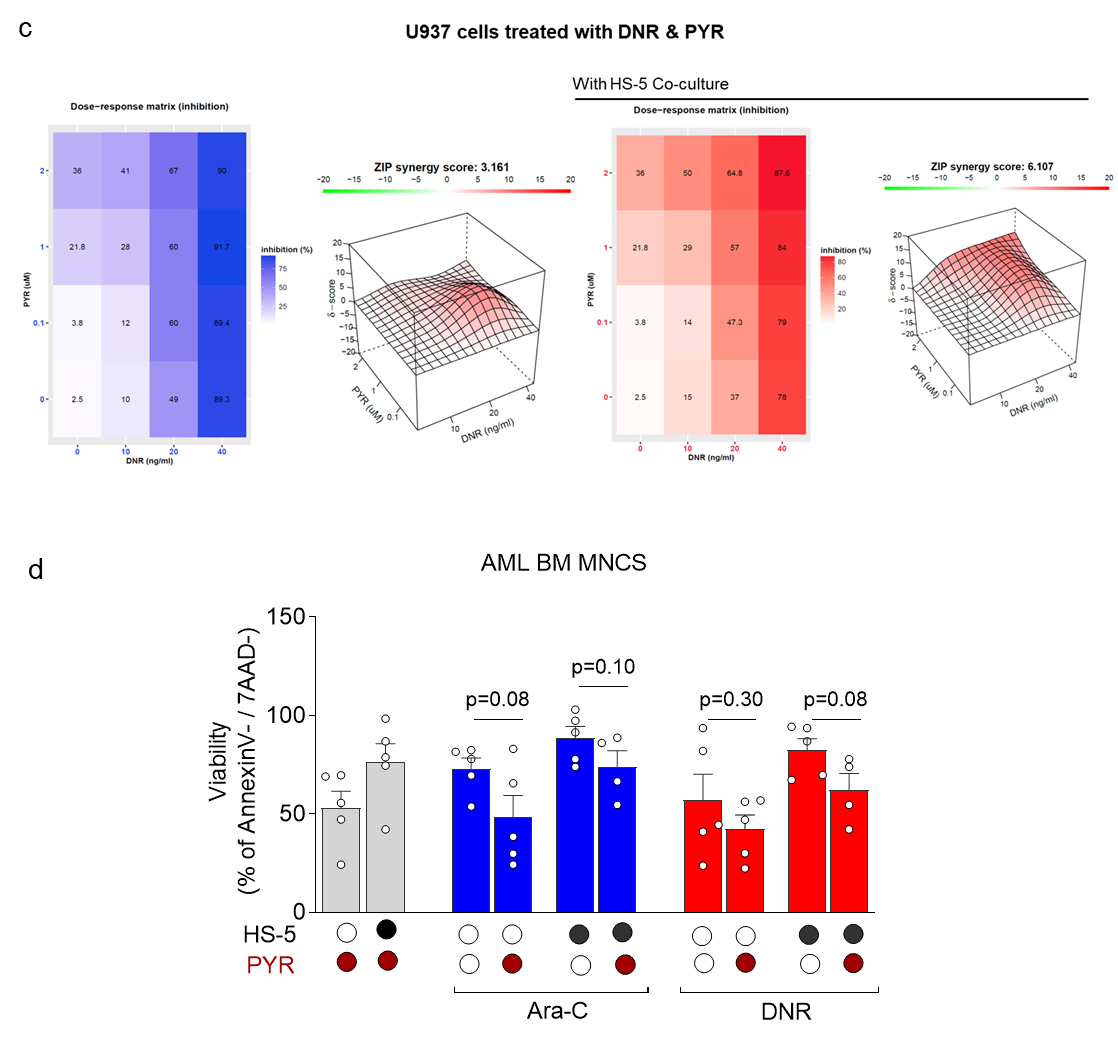
**

a) Synergy evaluation of ATO & PYR in NB4 cells by treating with increasing concentration of ATO (0.5, 1 and 2 µM) and PYR (0.1, 1 and 2 µM) with or without HS-5 co-culture for 48 hrs (n=4). Heatmap represents dose response matrix with % of inhibition value (100 - % viability) followed by distribution of synergy with synergy score. The synergy score was estimated using the ZIP (zero interaction potency) synergy model using the SynergyFinder tool. b) Synergy evaluation in U937 cells treated with Ara-C (100, 200 and 400ng/ml) and PYR with and without co-culture (n=3). c) Synergy assessment in U937 cells treated with DNR (10, 20, and 40ng/ml) and PYR with and without co-culture (n=3). d) Viability of primary leukemic cells AML BM MNCs upon co-culture with stromal cells treated with PYR in combination with Ara-C and DNR for 48 hrs (n=5).

**Supplementary figure 10:** **Comparing the effect of pyrvinium on pre/ post engraftment of leukemic cells and toxicity analysis of ATO+PYR in normal FVBN mice.**


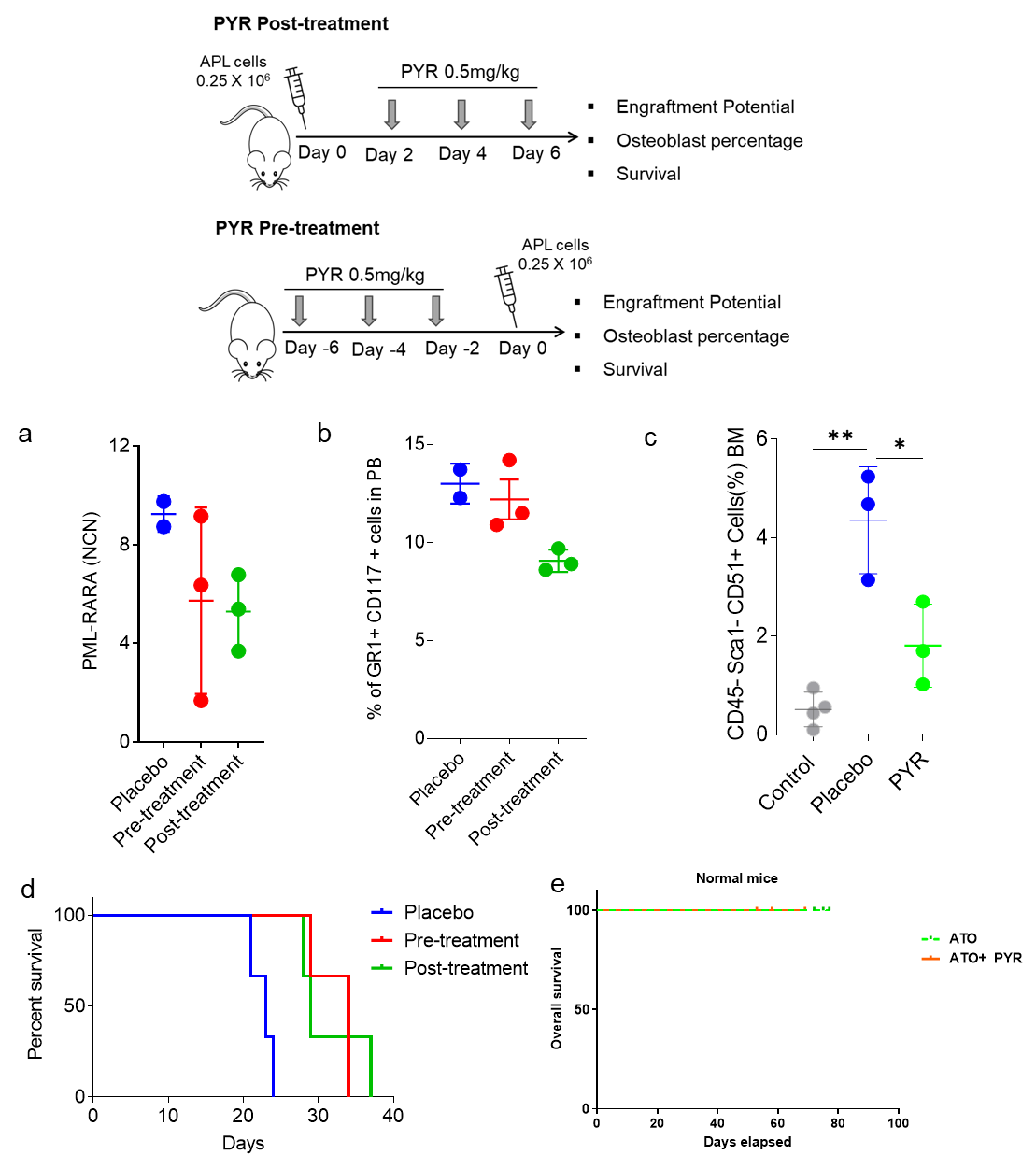


a) Q-PCR analysis revealed decreased tumor burden in APL mice as measured by PML-RARA normalized copy numbers (NCN) on day 21. b) Flow cytometric analysis of leukemic burden (GR1^+^ CD117^+^) cells in peripheral blood of mice on day 21. c) Flow cytometric analysis of osteoblast percentage (CD45^-^ Sca1^-^ CD51^+^) in mice treated with PYR at the time of death compared to placebo. d) Survival of mice on pre-treatment or post-treatment of Pyrvinium. e) survival of normal FVBN mice after administration of ATO (10mg/kg- 15 doses) and PYR (0.5mg/kg – 14 doses) like the dose given in the APL model *figure 6f*.

**Supplementary figure 11: Impact of lymphoid leukemic cells on differentiation of stromal cells.**


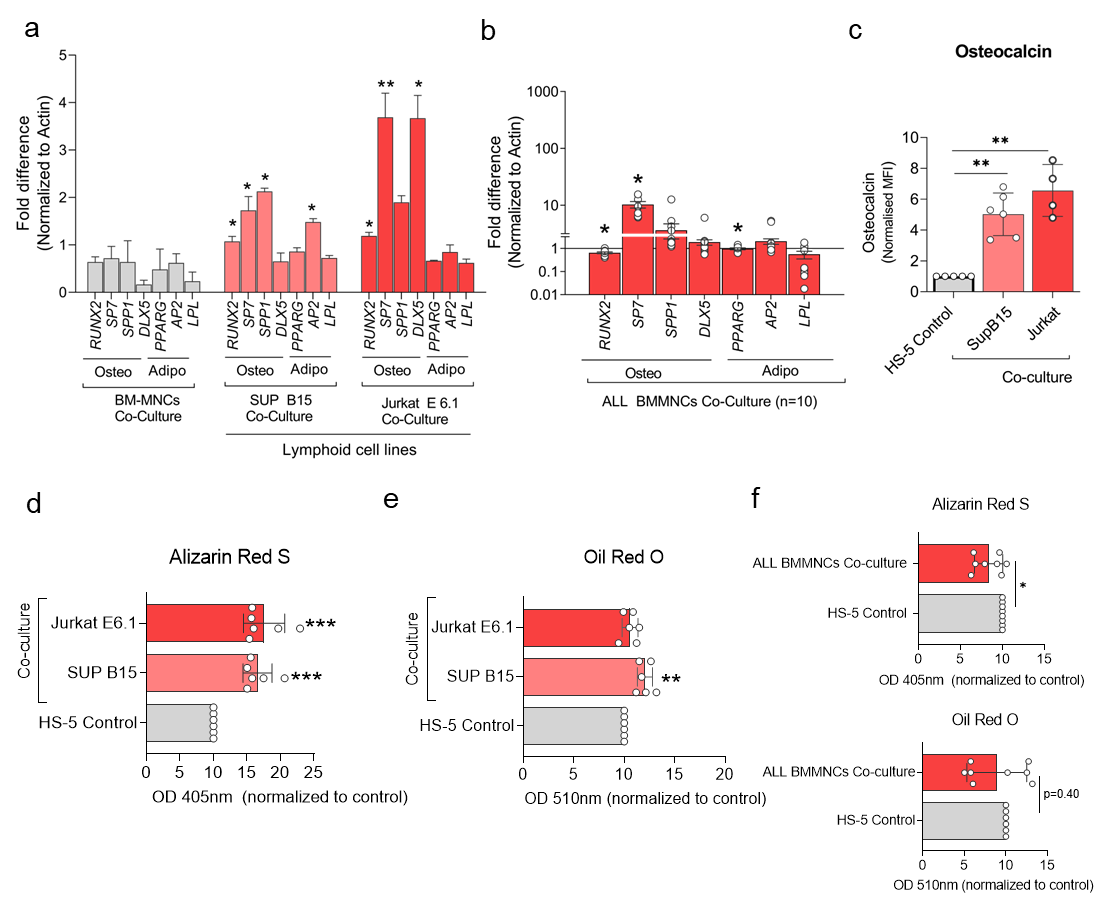


a) QPCR analysis of osteogenic (*RUNX2, SP7, SPP1 & DLX5*) and adipogenic genes *(PPARG, AP2 & LPL*) in stromal cells co-cultured with lymphoid leukemic cell lines (SUP B15 and Jurkat E6.1) cell lines in comparison to control BM-MNCs cells for 48 hrs through QPCR assay (n=4), followed by validation in stromal cells co-cultured with ALL BM-MNCs (n=10) shown in (b). c) Quantitative bar graph demonstrating osteocalcin's MFI in stromal cells after 48 hrs of co-culture with different leukemic cells. d) Differentiation of stromal cells into osteoblast after co-culturing with leukemic cells SUP B15 & Jurkat E6.1 cells for 48 hrs followed by differentiation of stromal cells was carried out for 14 days, and the cells were stained with Alizarin Red S staining. The colorimetric detection of Alizarin Red S staining using absorbance at 405nm (n=6). e) Adipocyte differentiation of stromal cells post leukemic cell co-culture followed by Oil Red O staining. The colorimetric detection of Oil Red O staining using absorbance at 510nm (n=6). f) Alizarin Red S (osteoblast differentiation) and Oil Red O staining (adipocyte differentiation) of stromal cells post 48 hrs of co-culture with primary ALL cells (n=8) followed by induction for 14 days.


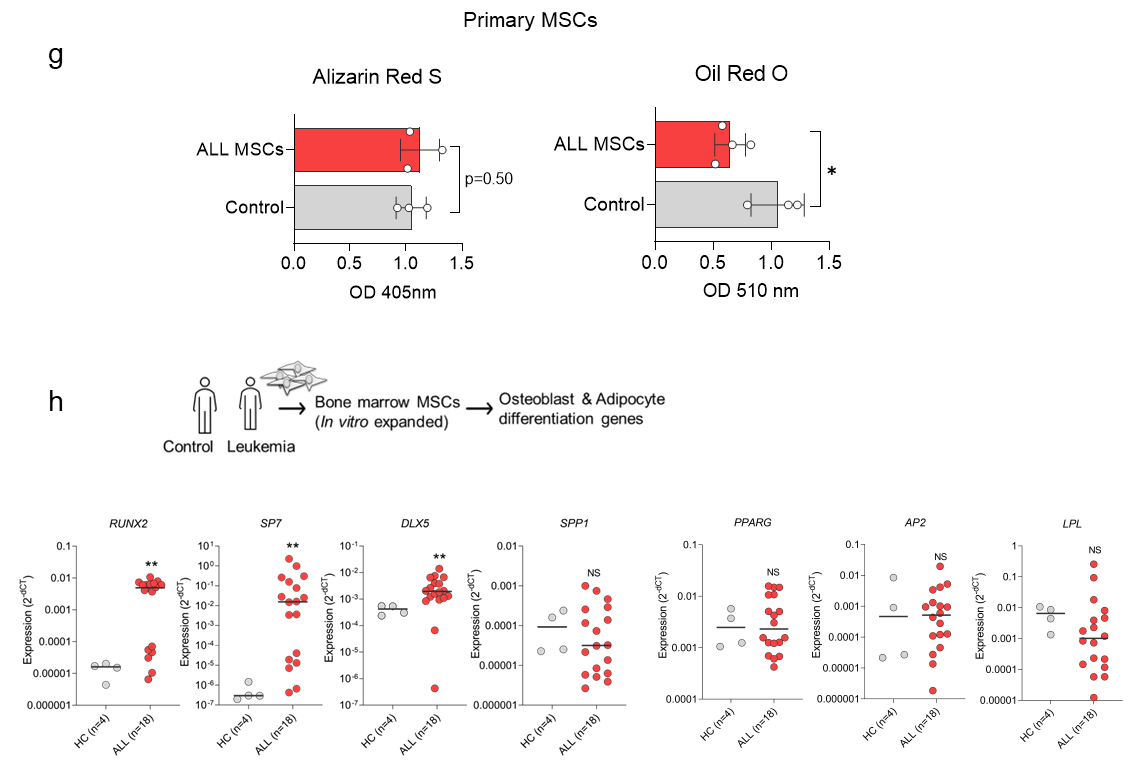


g) Primary bone marrow ALL MSCs illustrate the osteoblast and adipogenic differentiation potential through Alizarin Red S and Oil Red O staining (n=3). h) QPCR analysis of osteogenic and adipogenic genes in primary stromal cells expanded from the bone marrow samples of healthy control (HC (n=4)) ALL (n=18).

**Supplementary figure 12: Evaluation of Wnt signaling in stroma upon lymphoid leukemic cells co-culture and ALL MSCs**


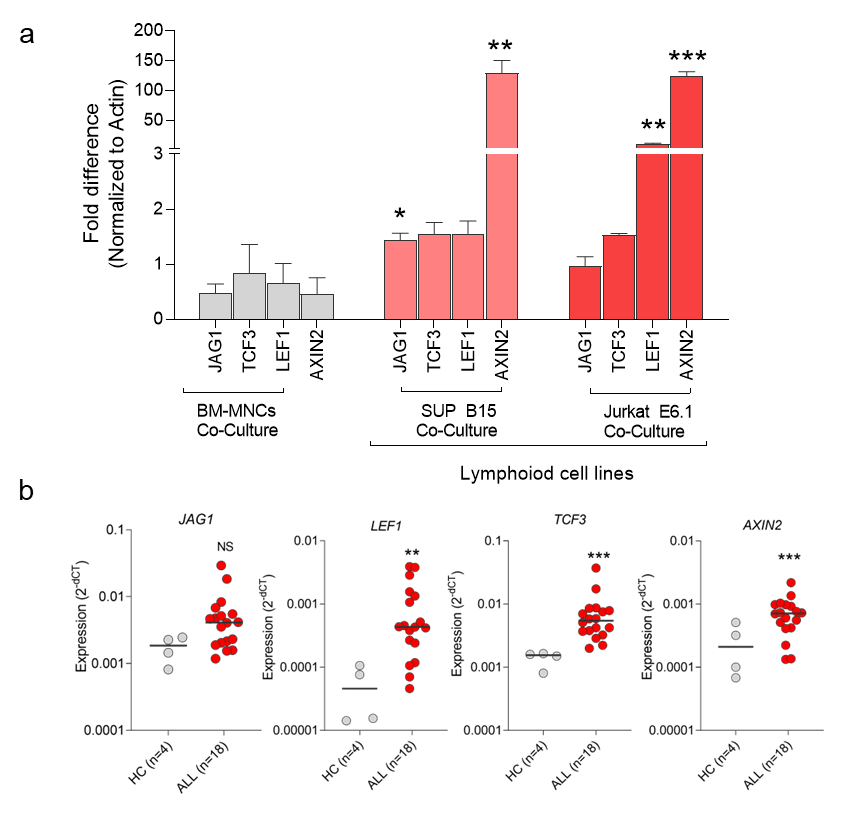


a) QPCR analysis of Wnt signaling genes in stromal cells (HS-5) post-co-culture with lymphoid leukemic cell lines SUP B15 and Jurkat E6.1 for 48 hrs (n=3). b) QPCR of Wnt signaling genes in primary MSCs expanded from the bone marrow of ALL (n=18) in comparison to HC (n=4).

**Supplementary figure 13: Chemoprotective effect of differentiated stromal cells on co-culture with lymphoid leukemic cells.**

**
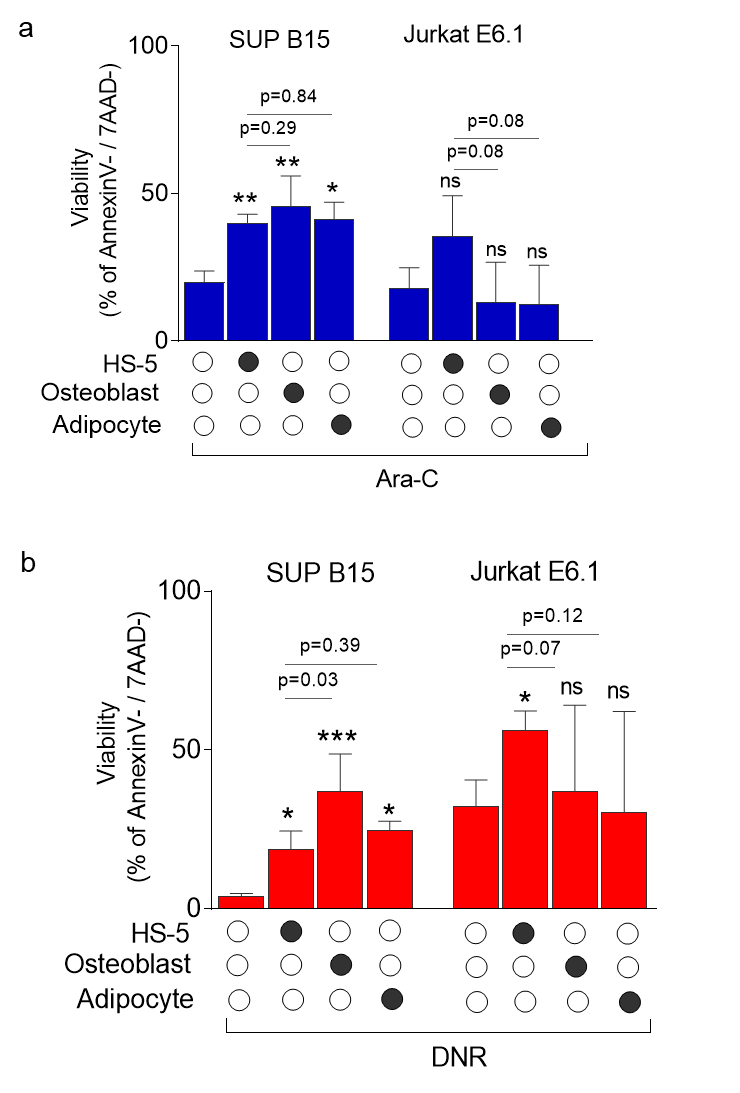
**

a) Viability of lymphoid leukemia cells (SUP B15 and Jurkat E6.1) upon co-culture with osteoblast and adipocyte differentiated stromal cells followed by treatment with cytarabine (Ara-C) at concentration compared to control. The viability was assessed after 48 hrs using annexin V/ 7AAD staining by flow cytometry (n=5). b) Viability of leukemic cells upon co-culture with stromal cells and followed by treatment with daunorubicin (DNR) (n=5). The viability of leukemic cells treated with the drug alone was compared with leukemic cells co-culture with different feeder layers, followed by comparison with osteoblast differentiated and undifferentiated cells.

**Supplementary figure 14: Pyrvinium pre-treatment in stromal cells reduces the drug resistance phenotype in lymphoid leukemic cells.**


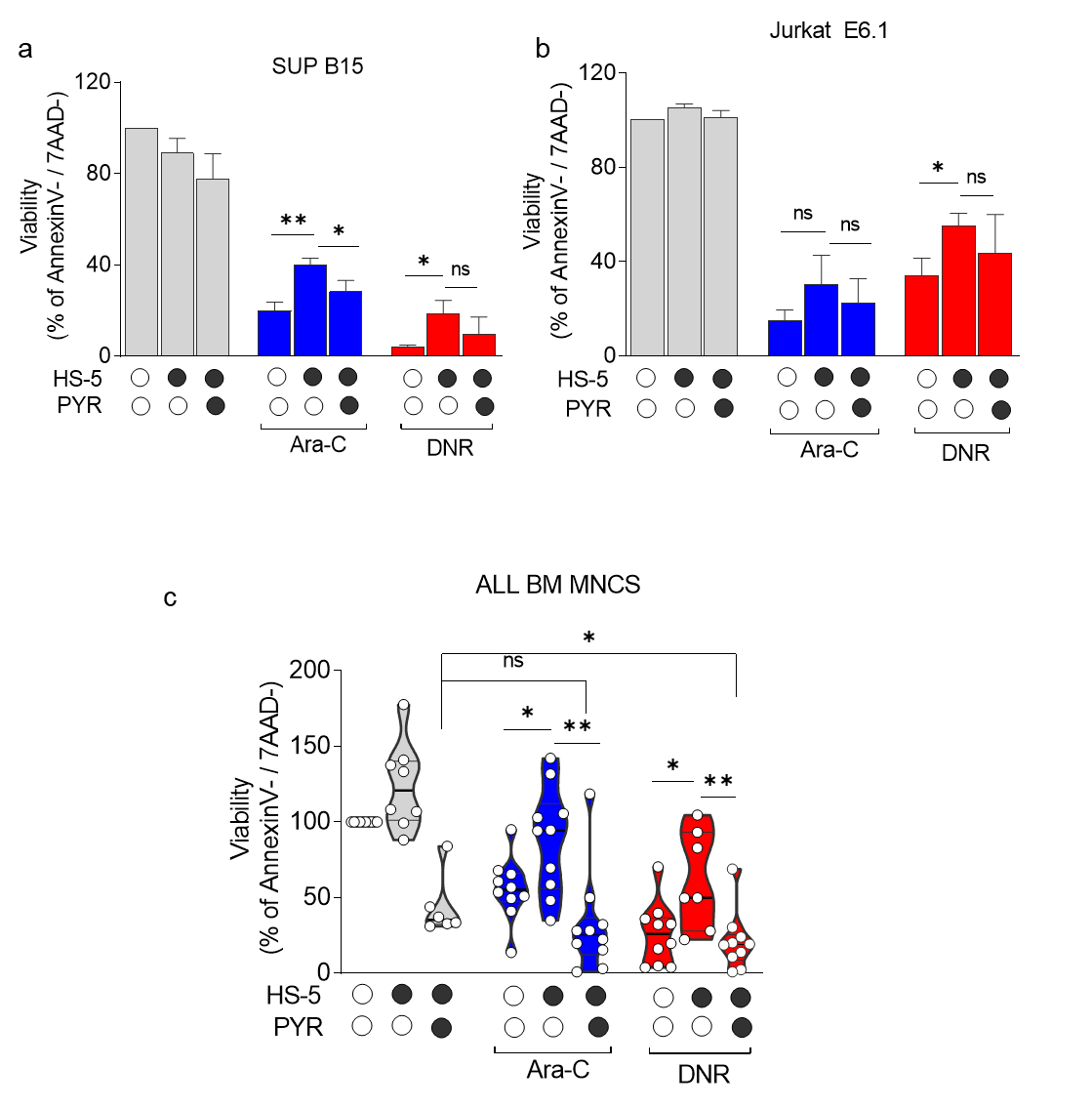


a) and b) Viability of lymphoid leukemic cells (SUP B15 and Jurkat E6.1) demonstrate that the PYR pre-treatment overcomes the stromal cell-mediated drug resistance in leukemic cells upon treatment with Ara-C and DNR (n=5). c) PYR pre-treatment reverses the drug resistance even in ALL BM MNCS (n=10), validating the cell line data.

**Supplementary figure 15. Full-length western blot images**


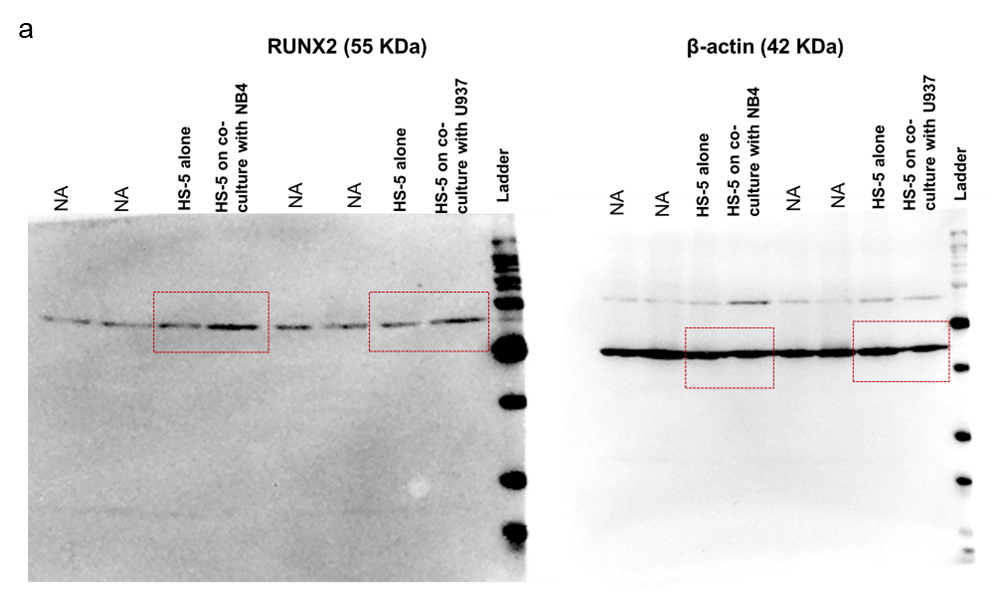


a) Immunoblot demonstrates an increased expression of *RUNX2* in the stromal cells co-cultured with leukemic cells (NB4 and U937) for 48 hrs. The same blot was stripped and probed for β-actin. *Main figure 2b*

**
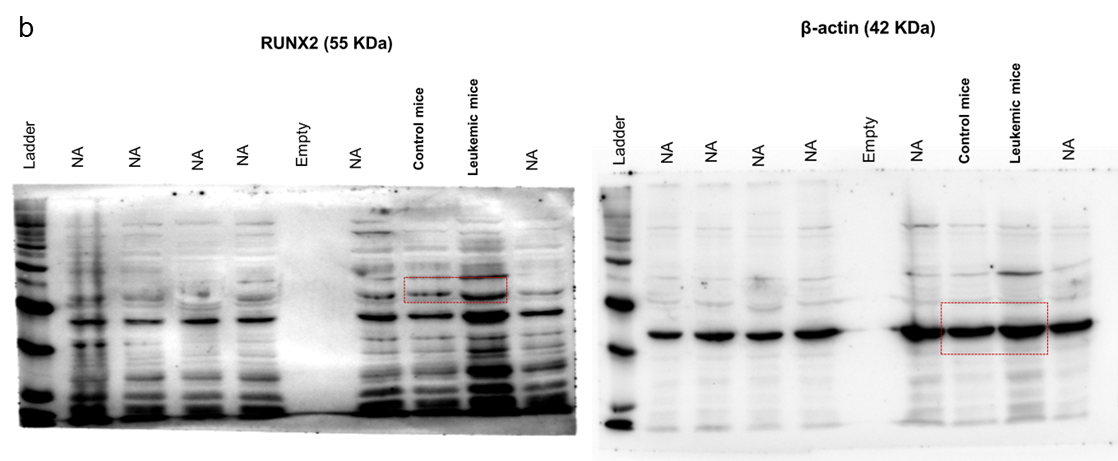
**

b) Immunoblot demonstrates an increased expression of *RUNX2* in leukemic mice compared to the control. *Main Figure 3b.*

**
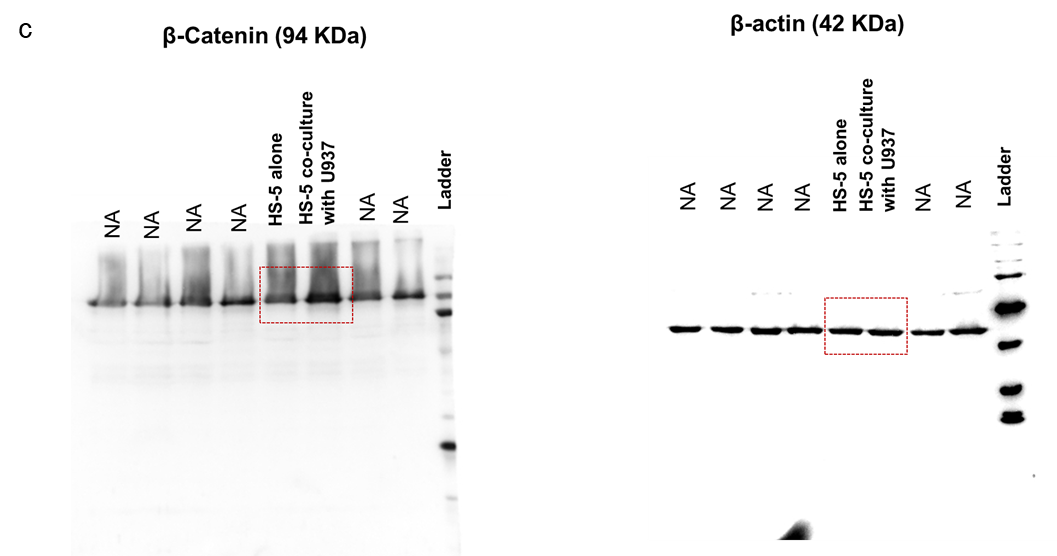
**

c) Immunoblot showing stabilization of β-catenin protein in the stromal cells upon co-culture with leukemic cells (U937) for 48 hrs. *Main Figure 5c*.

**
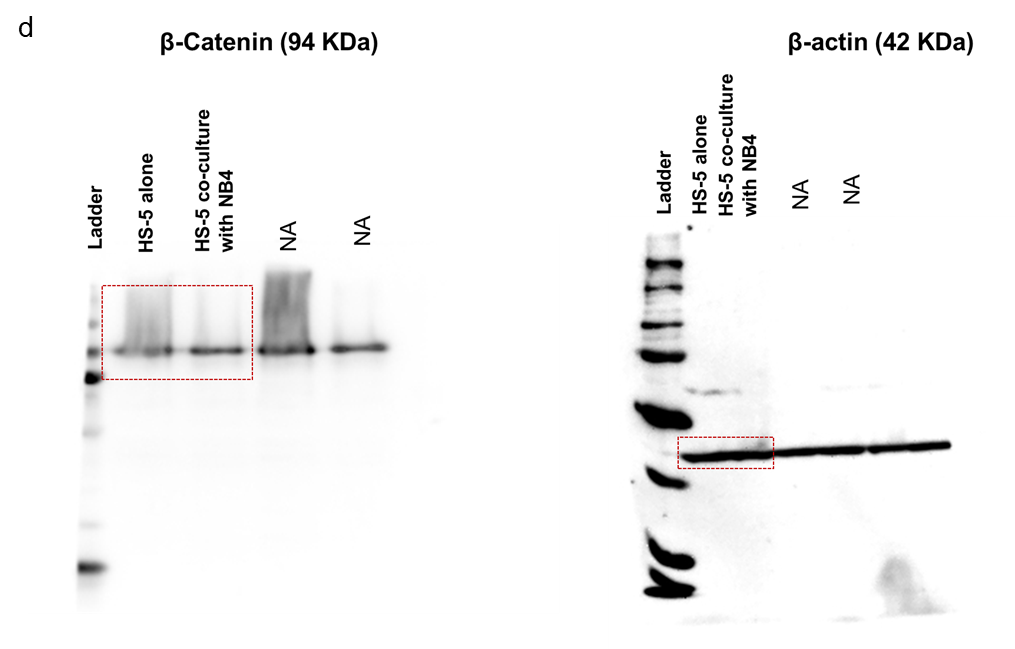
**

d) Immunoblot showing stabilization of β-catenin protein in the stromal cells upon co-culture with leukemic cells (NB4) for 48 hrs. *Main Figure 5c*.

**
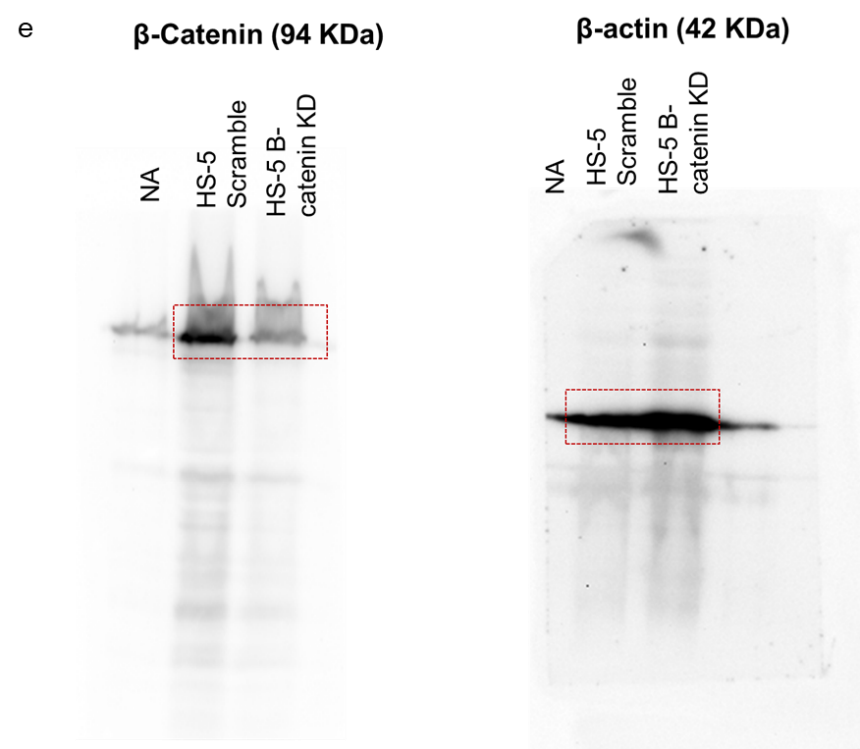
**

e) Immunoblot analysis demonstrating the knockdown (KD) efficacy of β-catenin in stromal cells HS-5 compared to scramble control. *Main Figure 6a.*

**Supplementary table S1**

**List of primer sequences used in this study.**

| **S. No** | **GENE NAME** | **PRIMER SEQUENCES** |
| --- | --- | --- |
|  | **Human** | |
| 1 | ACTB - Forward | 5' - CCCTAAGGCCAACCGTGAA - 3' |
| 2 | ACTB - Reverse | 5' - CCAGAGGCATACAGGGACAAC - 3' |
| 3 | RUNX2 - Forward | 5' - AAGTGCGGTGCAAACTTTCT - 3' |
| 4 | RUNX2 - Reverse | 5' - TCTCGGTGGCTGCTAGTGA - 3' |
| 5 | SPP1 - Forward | 5' - ACTCGAACGACTCTGATGATGT - 3' |
| 6 | SPP1 - Reverse | 5' - GTCAGGTCTGCGAAACTTCTTA - 3' |
| 7 | DLX5 - Forward | 5' - GTCTTCAGCTACCGATTCTGAC - 3' |
| 8 | DLX5 - Reverse | 5' - CTTTGCCATAGGAAGCCGAG - 3' |
| 9 | SP7 - Forward | 5' - GAGGCAACTGGCTAGGTGG - 3' |
| 10 | SP7 - Reverse | 5' - CTGGATTAAGGGGAGCAAAGTC - 3' |
| 11 | TCF3 - Forward | 5' - CCGACTCCTACAGTGGGCTA - 3' |
| 12 | TCF3 - Reverse | 5' - CGCTGACGTGTTCTCCTCG - 3' |
| 13 | JAG1 - Forward | 5' - GTCCATGCAGAACGTGAACG - 3' |
| 14 | JAG1 - Reverse | 5' - GCGGGACTGATACTCCTTGA - 3' |
| 15 | LEF1 - Forward | 5' - TGCCAAATATGAATAACGACCCA - 3' |
| 16 | LEF1 - Reverse | 5' - GAGAAAAGTGCTCGTCACTGT - 3' |
| 17 | PPARG - Forward | 5' - GCTGGCCTCCTTGATGAATA - 3' |
| 18 | PPARG - Reverse | 5' - TTGGGCTCCATAAAGTCACC - 3' |
| 19 | AP2 - Forward | 5' - AACCTTAGATGGGGGTGTCC - 3' |
| 20 | AP2 - Reverse | 5' - GTGGAAGTGACGCCTTTCAT - 3' |
| 21 | LPL - Forward | 5' - GGGCATGTTGACATTTACCC - 3' |
| 22 | LPL - Reverse | 5' - GCTGGTCCACATCTCCAAGT - 3' |
| 23 | AXIN2 - Forward | 5' - TACACTCCTTATTGGGCGATCA - 3' |
| 24 | AXIN2 - Reverse | 5' - TTGGCTACTCGTAAAGTTTTGGT - 3' |
|  | **Mouse** | |
| 25 | ACTB- Forward | 5' – GGCTGTATTCCCCTCCATCG - 3' |
| 26 | ACTB - Reverse | 5' – CCAGTTGGTAACAATGCCATGT - 3' |
| 27 | TCF1 - Forward | 5' – CAGAATCCACAGATACAGCA - 3' |
| 28 | TCF1 - Reverse | 5' – CAGCCTTTGAAATCTTCATC - 3' |
| 29 | LEF1 - Forward | 5' – AGTGCAGCTATCAACCAGAT - 3' |
| 30 | LEF1 - Reverse | 5' – TTCATAGTATTTGGCCTGCT - 3' |
| 31 | AXIN2 - Forward | 5' – ATGGAGTCCCTCCTTACCGCAT - 3' |
| 32 | AXIN2 - Reverse | 5' – GTTCCACAGGCGTCATCTCCTT - 3' |
| 33 | JAG1 - Forward | 5' – TGCGTGGTCAATGGAGACTCCT - 3' |
| 34 | JAG1 - Reverse | 5' – TCGCACCGATACCAGTTGTCTC - 3' |
| 35 | TCF3 - Forward | 5' – CCATGCTAGGTGACGGCTCTTC - 3' |
| 36 | TCF3 - Reverse | 5' – GCGAGCCATTAACCTCAGATCC - 3' |

**Supplementary table S2**

**Differential expression of genes associated with osteoblast differentiation from RNA sequencing analysis of stromal cells (HS-5) co-cultured with leukemic cells (U937)**

| **Gene** | **Locus** | **FPKM**  **(HS-5)** | **FPKM**  **(HS-5 co-culture)** | **log2(Fold change)** |
| --- | --- | --- | --- | --- |
| AHSG | chr3:186330849-186339107 | 0 | 0 | 0 |
| BMP5 | chr6:55620237-55740375 | 0 | 0 | 0 |
| COMP | chr19:18893582-18902114 | 0 | 0.0102265 | inf |
| DLX5 | chr7:96649701-96654143 | 0 | 0.055923 | inf |
| FGFR2 | chr10:123237843-123357972 | 0 | 0 | 0 |
| FN1 | chr2:216225178-216300791 | 0 | 0 | 0 |
| GDF10 | chr10:48425787-48439138 | 0 | 0 | 0 |
| SP7 | chr12:53720359-53730004 | 0 | 0 | 0 |
| TNFSF11 | chr13:43136871-43182149 | 0 | 0 | 0 |
| BGLAP | chr1:156182778-156213123 | 0 | 0 | 0 |
| DLX5 | chr7:96649701-96654143 | 0 | 0.055923 | inf |
| MSX2 | chr5:174151574-174157902 | 0 | 0 | 0 |
| DLX6 | chr7:96597826-96643377 | 0.0137878 | 0.0821803 | 2.5754 |
| TNF | chr6_ssto_hap7:2874150-2876913 | 0.0156882 | 0.0623393 | 1.99046 |
| CALCR | chr7:93053798-93204042 | 0.00688316 | 0.0205283 | 1.57647 |
| VCAM1 | chr1:101185195-101204601 | 0.00851568 | 0.0253822 | 1.57562 |
| COL2A1 | chr12:48366747-48398285 | 0.00996465 | 0.028446 | 1.51334 |
| MMP8 | chr11:102582525-102595685 | 0.0732327 | 0.177821 | 1.27987 |
| SPP1 | chr4:88896801-88904563 | 0.404101 | 0.892625 | 1.14334 |
| FLT1 | chr13:28874482-29069265 | 0.0695019 | 0.152055 | 1.12947 |
| COL10A1 | chr6:116421998-116566853 | 2.33301 | 3.8724 | 0.731036 |
| COL15A1 | chr9:101706137-101833068 | 0.0413892 | 0.0639523 | 0.627742 |
| TGFB2 | chr1:218517537-218617961 | 3.09625 | 4.73672 | 0.613366 |
| GLI1 | chr12:57853917-57873633 | 0.310598 | 0.468865 | 0.594126 |
| IGF2 | chr11:2150341-2182439 | 1.19619 | 1.72745 | 0.530202 |
| MMP9 | chr20:44637546-44645200 | 0.28203 | 0.398687 | 0.499411 |
| PHEX | chrX:22050920-22266478 | 0.192007 | 0.268754 | 0.485133 |
| TGFB1 | chr19:41836811-41859831 | 32.2496 | 42.9713 | 0.414093 |
| COL14A1 | chr8:121137351-121384273 | 0.335784 | 0.433594 | 0.368811 |
| CD36 | chr7:80231503-80308593 | 0.410162 | 0.512264 | 0.320695 |
| CTNNBIP1 | chr1:9908333-9970316 | 3.50398 | 4.28613 | 0.290682 |
| CTSK | chr1:150768683-150780917 | 157.966 | 191.628 | 0.278702 |
| TGFBR1 | chr9:101867411-101916473 | 12.1803 | 14.7241 | 0.273637 |
| ITGA3 | chr17:48133339-48167849 | 45.5704 | 54.5378 | 0.259159 |
| ICAM1 | chr19:10381516-10397291 | 72.3974 | 86.489 | 0.256579 |
| CDH11 | chr16:64980682-65155919 | 1.23529 | 1.44161 | 0.222834 |
| BGN | chrX:152760346-152775004 | 6.43629 | 7.50191 | 0.22103 |
| ITGA11 | chr15:68594041-68724492 | 3.45315 | 2.12933 | -0.69752 |
| CSF1 | chr1:110453232-110473616 | 32.7332 | 37.2743 | 0.18743 |
| VEGFB | chr11:64002055-64006736 | 32.3586 | 35.7629 | 0.144316 |
| EGF | chr4:110834039-110934118 | 0.208723 | 0.223923 | 0.101409 |
| TWIST1 | chr7:19155090-19157295 | 13.3461 | 13.8672 | 0.055259 |
| ITGA2 | chr5:52285155-52390609 | 22.7143 | 23.5668 | 0.053158 |
| BMP6 | chr6:7727010-8102828 | 1.68824 | 1.73258 | 0.037404 |
| CHRD | chr3:184097860-184107617 | 2.46624 | 2.52634 | 0.034739 |
| COL5A1 | chr9:137533651-137736688 | 53.7013 | 54.9278 | 0.032578 |
| BMPR1B | chr4:95679127-96079601 | 1.01007 | 1.02925 | 0.027147 |
| SP3 | chr2:174771186-174830430 | 22.8311 | 23.2424 | 0.025757 |
| SMAD5 | chr5:135465202-135518422 | 17.7526 | 18.0313 | 0.022473 |
| COL1A2 | chr7:94023872-94060544 | 736.383 | 744.895 | 0.01658 |
| SMAD2 | chr18:45359465-45457515 | 7.85672 | 7.93498 | 0.014299 |
| MSX1 | chr4:4861391-4865660 | 11.1744 | 11.2723 | 0.012588 |
| ITGB1 | chr10:33189245-33247293 | 178.564 | 179.84 | 0.010279 |
| FGF2 | chr4:123747862-123844159 | 73.8923 | 74.3499 | 0.008906 |
| ATF4 | chr22:39916568-39918691 | 197.561 | 196.976 | -0.00428 |
| NOG | chr17:54671059-54672951 | 0.327632 | 0.325467 | -0.00956 |
| IGF1 | chr12:102789644-102874378 | 0.0100047 | 0.00993763 | -0.0097 |
| CSF2 | chr5:131409484-131411863 | 22.1959 | 21.9399 | -0.01673 |
| TGFB3 | chr14:76424441-76448092 | 1.53532 | 1.5096 | -0.02437 |
| PDGFA | chr7:536896-559481 | 4.43596 | 4.35924 | -0.02517 |
| COL1A1 | chr17:48261456-48279000 | 260.418 | 255.605 | -0.02691 |
| IGF1R | chr15:99192760-99507759 | 4.93626 | 4.83997 | -0.02842 |
| FGF1 | chr5:141971742-142077635 | 0.741388 | 0.719228 | -0.04378 |
| BMPR2 | chr2:203241049-203432474 | 7.95931 | 7.61999 | -0.06285 |
| TGFBR2 | chr3:30647993-30735633 | 19.8094 | 18.8188 | -0.07401 |
| CSF3 | chr17:38171613-38174066 | 31.5023 | 29.8746 | -0.07654 |
| ACVR1 | chr2:158592957-158732374 | 20.7868 | 19.7011 | -0.07739 |
| BMPR1A | chr10:88516395-88684945 | 11.4253 | 10.8212 | -0.07837 |
| FGFR1 | chr8:38268655-38326352 | 31.6735 | 29.9013 | -0.08307 |
| SMAD4 | chr18:48556582-48611411 | 11.5102 | 10.8586 | -0.08407 |
| SMAD1 | chr4:146402950-146480325 | 6.90728 | 6.50932 | -0.08561 |
| MMP10 | chr11:102641232-102651359 | 0.251551 | 0.235192 | -0.09701 |
| SERPINH1 | chr11:75273100-75283849 | 146.484 | 135.679 | -0.11054 |
| ANXA5 | chr4:122589151-122618147 | 794.758 | 729.733 | -0.12315 |
| VEGFA | chr6:43737945-43754223 | 37.0661 | 33.9227 | -0.12785 |
| BMP1 | chr8:22022652-22069840 | 12.5493 | 11.4689 | -0.12987 |
| COL3A1 | chr2:189839098-189877472 | 172.018 | 155.181 | -0.14861 |
| MMP2 | chr16:55513080-55540586 | 207.1 | 186.571 | -0.1506 |
| ITGAM | chr16:31271287-31344213 | 0.0872683 | 0.0764614 | -0.19073 |
| EGFR | chr7:55086724-55275031 | 42.2814 | 36.9222 | -0.19553 |
| NFKB1 | chr4:103422485-103538459 | 22.9413 | 18.9387 | -0.27661 |
| BMP2 | chr20:6748744-6760910 | 5.12195 | 4.1094 | -0.31777 |
| SMAD3 | chr15:67358194-67487533 | 35.7172 | 28.2321 | -0.33928 |
| RUNX2 | chr6:44796469-45518819 | 5.82429 | 4.5806 | -0.34654 |
| IHH | chr2:219919141-219925238 | 0.0988513 | 0.0736477 | -0.42462 |
| BMP4 | chr14:54416454-54423554 | 0.873005 | 0.628079 | -0.47504 |
| SOX9 | chr17:70117160-70122560 | 2.36371 | 1.69744 | -0.47769 |
| VDR | chr12:48235319-48298814 | 12.0274 | 7.84136 | -0.61715 |
| ALPL | chr1:21835857-21904905 | 0.1143 | 0.0652844 | -0.80801 |
| BMP7 | chr20:55743808-55841707 | 0.0121597 | 0.00603922 | -1.00967 |

**Supplementary references**

1. Subramanian A, Tamayo P, Mootha VK, Mukherjee S, Ebert BL, Gillette MA, et al. Gene set enrichment analysis: a knowledge-based approach for interpreting genome-wide expression profiles. Proc Natl Acad Sci U S A. 2005 Oct 25;102(43):15545–50.

2. Ianevski A, Giri AK, Aittokallio T. SynergyFinder 3.0: an interactive analysis and consensus interpretation of multi-drug synergies across multiple samples. Nucleic Acids Research. 2022 Jul 5;50(W1):W739–43.
